# The topography of mutational processes in breast cancer genomes

**DOI:** 10.1038/ncomms11383

**Published:** 2016-05-02

**Authors:** Sandro Morganella, Ludmil B. Alexandrov, Dominik Glodzik, Xueqing Zou, Helen Davies, Johan Staaf, Anieta M. Sieuwerts, Arie B. Brinkman, Sancha Martin, Manasa Ramakrishna, Adam Butler, Hyung-Yong Kim, Åke Borg, Christos Sotiriou, P. Andrew Futreal, Peter J. Campbell, Paul N. Span, Steven Van Laere, Sunil R. Lakhani, Jorunn E. Eyfjord, Alastair M. Thompson, Hendrik G. Stunnenberg, Marc J. van de Vijver, John W. M. Martens, Anne-Lise Børresen-Dale, Andrea L. Richardson, Gu Kong, Gilles Thomas, Julian Sale, Cristina Rada, Michael R. Stratton, Ewan Birney, Serena Nik-Zainal

**Affiliations:** 1 European Molecular Biology Laboratory, European Bioinformatics Institute, Wellcome Trust Genome Campus, Cambridgeshire CB10 1SD, UK; 2 Wellcome Trust Sanger Institute, Cambridge CB10 1SA, UK; 3 Theoretical Biology and Biophysics (T-6), Los Alamos National Laboratory, Los Alamos NM 87545, New Mexico, USA; 4 Center for Nonlinear Studies, Los Alamos National Laboratory, Los Alamos NM 87545, New Mexico, USA; 5 Division of Oncology and Pathology, Department of Clinical Sciences Lund, Lund University, Lund SE-223 81, Sweden; 6 Department of Medical Oncology, Erasmus MC Cancer Institute and Cancer Genomics Netherlands, Erasmus University Medical Center, Rotterdam 3015CN, The Netherlands; 7 Radboud University, Faculty of Science, Department of Molecular Biology, 6525GA Nijmegen, The Netherlands; 8 Department of Pathology, College of Medicine, Hanyang University, Seoul 133-791, South Korea; 9 Breast Cancer Translational Research Laboratory, Université Libre de Bruxelles, Institut Jules Bordet, Bd de Waterloo 121, B-1000 Brussels, Belgium; 10 Department of Genomic Medicine, UT MD Anderson Cancer Center, Houston, Texas 77230, USA; 11 Department of Radiation Oncology, and department of Laboratory Medicine, Radboud university medical center, Nijmegen 6525GA, The Netherlands; 12 Translational Cancer Research Unit, GZA Hospitals Sint-Augustinus, Wilrijk, Belgium and Center for Oncological Research, University of Antwerp, Antwerp B-2610, Belgium; 13 Centre for Clinical Research and School of Medicine, University of Queensland, Brisbane, Queensland 4059, Australia; 14 Pathology Queensland, The Royal Brisbane and Women's Hospital, Brisbane, Queensland 4029, Australia; 15 Cancer Research Laboratory, Faculty of Medicine, University of Iceland, 101 Reykjavik, Iceland; 16 Department of Breast Surgical Oncology, University of Texas MD Anderson Cancer Center, 1400 Pressler Street,Houston, Texas 77030, USA; 17 Department of Surgical Oncology, University of Dundee, Dundee DD1 9SY, UK; 18 Department of Pathology, Academic Medical Center, Meibergdreef 9, 1105 AZ Amsterdam, The Netherlands; 19 Department of Cancer Genetics, Institute for Cancer Research, Oslo University Hospital, The Norwegian Radium Hospital, Oslo 0310, Norway; 20 K.G. Jebsen Centre for Breast Cancer Research, Institute for Clinical Medicine, University of Oslo, Oslo 0310, Norway; 21 Department of Pathology, Brigham and Women's Hospital, Boston, Massachusetts 02115, USA; 22 Department of Cancer Biology, Dana-Farber Cancer Institute, Boston, Massachusetts 02215, USA; 23 Synergie Lyon Cancer, Centre Léon Bérard, 28 rue Laënnec, Lyon Cedex 08, France; 24 MRC Laboratory of Molecular Biology, Francis Crick Avenue, Cambridge CB2 0QH, UK; 25 East Anglian Medical Genetics Service, Cambridge University Hospitals NHS Foundation Trust, Cambridge CB2 9NB, UK

## Abstract

Somatic mutations in human cancers show unevenness in genomic distribution that correlate with aspects of genome structure and function. These mutations are, however, generated by multiple mutational processes operating through the cellular lineage between the fertilized egg and the cancer cell, each composed of specific DNA damage and repair components and leaving its own characteristic mutational signature on the genome. Using somatic mutation catalogues from 560 breast cancer whole-genome sequences, here we show that each of 12 base substitution, 2 insertion/deletion (indel) and 6 rearrangement mutational signatures present in breast tissue, exhibit distinct relationships with genomic features relating to transcription, DNA replication and chromatin organization. This signature-based approach permits visualization of the genomic distribution of mutational processes associated with APOBEC enzymes, mismatch repair deficiency and homologous recombinational repair deficiency, as well as mutational processes of unknown aetiology. Furthermore, it highlights mechanistic insights including a putative replication-dependent mechanism of APOBEC-related mutagenesis.

## Abstract (web summary)

Mutational signatures provide evidence of the mechanism of action of a given mutagen and are found in cancer cells. Here, using 560 breast cancer genomes, the authors demonstrate that mutational signatures are frequently associated with genomic architecture including nucleosome positioning and replication timing.

## Graphical abstract

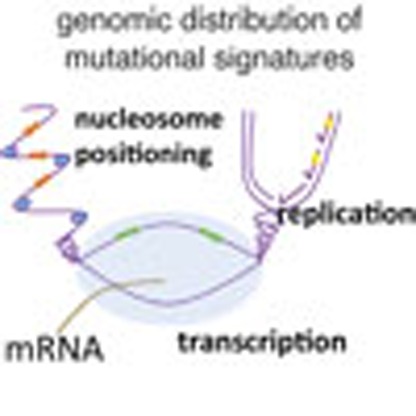

Correlations between the density of somatic mutations and various features of genomic structure and function have customarily been performed on aggregated cancer mutations across many cancer types^1^,^2^,^3^,^4^,^5^,^6^,^7^,^8^,^9^. These reports show similar general conclusions, for example, that substitution mutations are enriched in genomic regions that undergo replication late while rearrangements are enriched in early replicating regions^1^,^2^,^3^,^4^,^5^,^6^,^7^,^8^,^9^ or that specific genomic landmarks like chromatin organization are variably associated with mutation distribution^9^,^10^.

The interpretation of these historic analyses is, however, complicated, because somatic mutations do not arise from a single, universal mutagenic process. They occur due to numerous mutational processes that have occurred throughout the lifetime of the cancer patient^11^,^12^,^13^,^14^ and may be distinct in different tissues. Consider analyses based on simple substitution classes across multiple cancers. C>T transitions, for example, could arise from disparate mutational processes including deamination of methylated cytosines, deamination by APOBEC cytidine deaminases, exposure to ultraviolet irradiation or mismatch repair (MMR) deficiency. The interpretation of how C>T mutations are distributed relative to any genomic landmark would thus be limited by the complexity of mutational processes that contribute to C>T mutations.

In addition, previous analyses commonly combined data across several cancer types with diverse tissues of origin. However, exposures to DNA-damaging agents are likely to be different between tissues (for example, ultraviolet damage occurs in skin but not colorectal tissue) and DNA repair pathways may behave differently in cells of different organs. Moreover, replicative, transcriptional and chromatin dynamics may be distinct from one tissue to another, further hampering interpretation of such aggregated somatic mutation data^10^.

Each mutational process will leave its own specific pattern on the genome or mutational signature^11^ regardless of whether it arose as a pre-neoplastic process or post-malignant transformation. Recent advances in the mathematical extraction of mutational signatures^14^ from cancer sequences have led to the discovery of 21 such signatures in 30 different cancer types^14^. In a recent article of 560 highly curated whole-genome sequenced (WGS) breast cancers^15^, we extracted 12 base substitution mutational signatures from 3,479,652 base substitutions (signatures 1, 2, 3, 5, 6, 8, 13, 17, 18, 20, 26 and 30). These signatures were based on a 96-mutation classification that incorporates the base substitution type (expressed as the pyrimidine of a mutated Watson–Crick base pair, C>A, C>G, C>T, T>A, T>C, T>G) and the immediate flanking sequence context of the mutated base (four possible 5′ and four possible 3′ bases)^11^,^14^. We also analysed 77,695 rearrangements that were classified according to rearrangement type (deletions, tandem duplications, inversions and translocations), size (range 1 kilobase to >1 Mb) and whether they were focal or genomically dispersed, to extract six novel rearrangement signatures (RS1–RS6)^15^. These had different predominating features including being mainly characterized by tandem duplications (RS1 and RS3), deletions (RS5), clustered rearrangements (RS2, RS4) or translocations (RS2). In addition, 371,993 indels were categorized into two distinct signatures. ‘Repeat-mediated' deletions share the identical motif as a flanking polynucleotide repeat tract, are small (<3 bp) and arise from erroneous repair of insertion–deletion loops at polynucleotide tracts, the onus of post-replicative MMR^16^. In contrast, microhomology-mediated deletions show homology of several nucleotides between the start of the deletion and the flanking sequence of the deletion junction. They are usually larger (≥3 bp) than repeat-mediated deletions and are associated with repair by microhomology-mediated end joining mechanisms.

The significance of these signatures is clear. They are a proxy for the biological processes that have gone awry in breast tissue (see Table 1 for summary of signatures, their characteristics and putative aetiologies). Some associations include homologous recombination (HR) repair deficiency with signatures 3 and 8, microhomology-mediated indels, RS1, RS3 and RS5, putative activity of the APOBEC family of cytidine deaminases with signatures 2 and 13, MMR deficiency with signatures 6, 20 and 26 and an excess of repeat-mediated indels and deamination of methylated cytosines with signature 1. Aetiologies of the remaining signatures (signatures 5, 17, 18, 30; RS2, RS4 and RS6) are currently unknown (Table 1). This set of 560 breast cancers is the largest cohort of WGS cancers of a single tissue-type to date providing an exceptional opportunity to gain insights into mutagenic processes of a specific tissue.

We thus set out to comprehensively explore how mutation signatures in human breast cancers are influenced by genomic architecture. Critically, we were able to assign probabilistic estimates for individual somatic mutations to each mutational signature for every sample. Thus, by studying the genomic distribution of mutations as mutational signatures, we are able to interpret how mutagenic processes in breast tissues are influenced by cellular activities such as replication, transcription or by physical features like nucleosome occupancy.

## Results

### Diverse temporal relationships with replication

**Figure 1:**
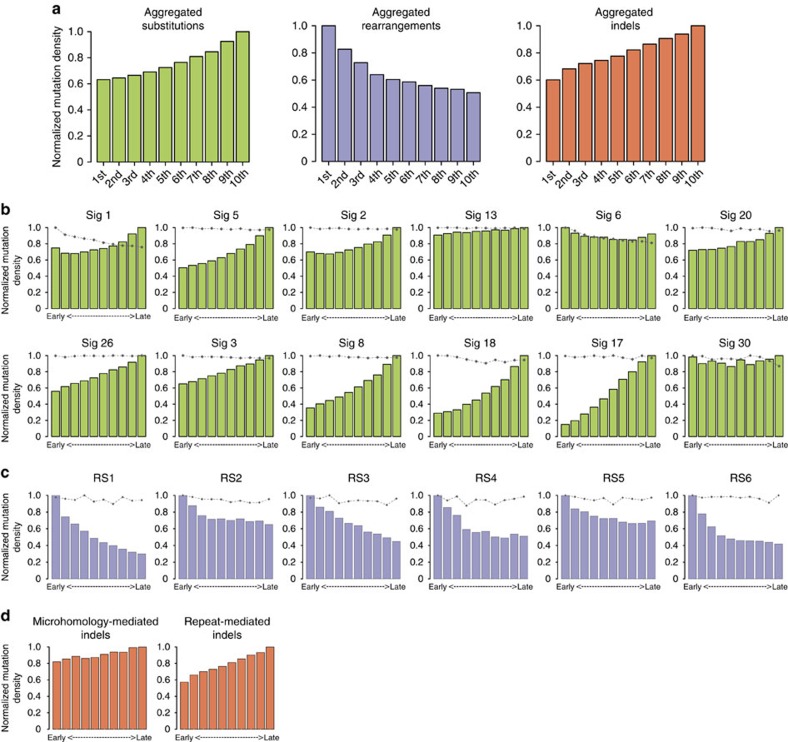
Distribution of all mutations across the cell cycle. Replication domains were identified by using conservatively defined transition zones in DNA replication time data. Data were separated into deciles, with each segment containing exactly 10% of the observed replication time signal. Normalized mutation density per decile is presented for early (left) to late (right) replication domains. (**a**) Aggregated distribution of mutations (green), rearrangements (purple) and indels (orange) across the cell cycle. (**b**) Distribution of the 12 base substitution signatures across the cell cycle. Dashed grey lines represent the predicted distribution of mutations for each signature based on simulations that take into account mutation burden and sequence characteristics of individual mutations and of the signatures that were estimated to be present in each patient (Methods section). (**c**) Distribution of the six rearrangement signatures across the cell cycle. Dashed grey lines represent the predicted distribution of mutations for each signature based on simulations. (**d**) Distribution of the indel signatures across the cell cycle.

DNA replication begins at origins (or near clusters of origins) of replication and propagates bidirectionally from each starting point^17^,^18^ with some regions of the genome copied sooner (early replicating) than others (late replicating)^19^. Using replication-sequencing (Repli-Seq)^20^ data generated from the breast cancer cell line, MCF-7 (ref. 20), early- and late-replicating regions were determined (Supplementary Fig. 1a–b) and relationships between mutations attributed to each signature and replication time were explored (Fig.1, Supplementary Fig. 1c–e).

Base-substitution signatures 1, 2, 3, 5, 8, 17, 18, 20 and 26 showed increases in mutation density from early to late replication, in keeping with previously described observations on aggregated substitutions. However, each had a distinctive gradient (Fig. 1b, Supplementary Fig. 1c–e) underscoring the individuality of each signature. In contrast, signature 6, 13 and 30 showed the unexpected tendency of relatively constant mutation densities through all replication time domains.

All six rearrangement signatures (RS1–RS6) from the 560 breast cancers^15^ were enriched in early replication. However, the gradient of change from early to late replication was variable between them (Fig. 1c). There was an approximately twofold reduction in rearrangement frequencies between the earliest and latest replication domains for RS1, RS3, RS4 and RS6. In contrast, RS2 and RS5 had flatter gradients with a greater proportion of rearrangements found in late replication domains than the other rearrangement signatures.

Somatic deletions were generally enriched late in replication. Repeat-mediated deletions showed a steep gradient with more mutations in late-replication time domains. Ten cancers with overwhelming indel mutagenesis (range 2,535–66,764) associated with MMR deficiency demonstrated a particularly steep gradient. In contrast, microhomology-mediated deletions demonstrated a gradual slope of increasing frequency towards late replication domains (Fig. 1d).

Thus, the signature-based approach permits higher resolution observations of distinctive variation between different mutational processes including behaviours different from those found when all mutations are considered together.

### Direction of replication influences mutational distribution

**Figure 2:**
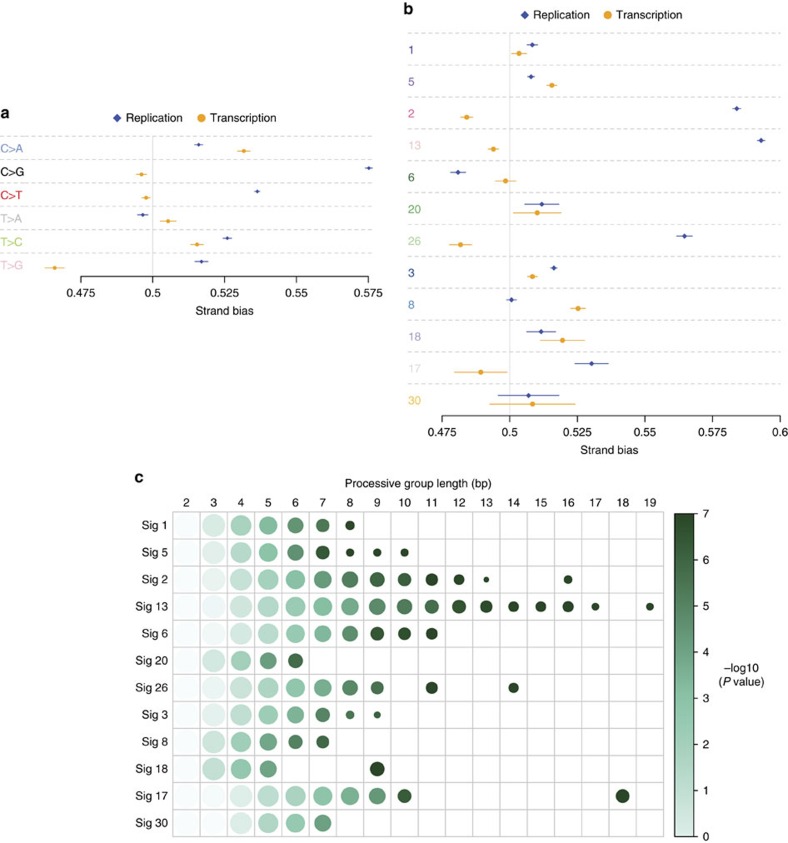
Replication and transcriptional strand bias and strand-coordinated mutagenesis of mutational signatures. Forest plots showing replication (blue) and transcription (orange) strand bias for the 6 base substitution classes (**a**) and for the 12 base substitution signatures (**b**). Mutations were oriented in the pyrimidine context (the current convention for characterizing mutational signatures). Observed distribution between strands is shown as a diamond for replication and circle for transcriptional strands with 95% confidence intervals, against an expected probability of 0.5 (Supplementary Table 1 for values). (**c**) Relationship between processive group lengths (columns) and mutational signatures (rows). Processive groups were defined as sets of adjacent substitutions of the same mutational signature sharing the same reference allele, and the group length indicates the number of adjacent substitutions within each group. The size of each circle represents the number of groups (log10) observed for the specified group length (column) for each signature (row). The intensity of colour of each circle indicates significance of the likelihood of detection of a processive group of a defined length (−log10 of the *P* value obtained by comparing observed data to simulations, further details in Methods section).

Replication fork migration from early to late replicative regions generates replicative strands that act as templates for DNA synthesis in a continuous and discontinuous manner, respectively^18^ (Supplementary Fig. 2a). Through knowing the direction of replication fork migration relative to the p-to-q orientation of the genome, transition zones could be assigned to p-to-q leading or p-to-q lagging replicative strands, respectively (Methods section, Supplementary Fig. 2b–d). We were conservative in our assignments excluding the first and last 25 kb of the latest replication domains and discarding regions of 10 kb or less indicative of where potential replicative strand switches could have occurred^21^,^22^ (Methods section). We thus explored whether the direction of replication influenced mutational processes through differences in mutation distribution between replicative strands (Fig. 2a,b, Supplementary Fig. 2e–i).

The level of asymmetry between strands is referred to as strand imbalance. A strand imbalance of 20% implies that one strand has 20% more mutations than the other (for example, for every 100 mutations on one strand, there are 120 on the other). We found that the level of asymmetry was different between the various base substitution signatures (Fig. 2b, Supplementary Table 1). Signatures 2, 13 and 26 exhibited strong replicative strand asymmetries with imbalances >30% for each of the signatures (*P* value <2.2e^−16^); signatures 1, 3, 5, 6, 17, 18 and 20 had weaker asymmetries (*P* value <2e^−4^ and strand imbalance <13%) and signatures 8, and 30 did not exhibit asymmetries of distribution between replication strands (*P* value >0.01 and strand imbalance <3%). Asymmetric mutation distribution between replicative strands was thus observed more markedly for a variety of different biological processes including putative APOBEC-related mutagenesis (signatures 2 and 13), and MMR deficiency (signatures 6, 20 and 26—all with different biases), implying that the direction of travel of a replication fork can influence somatic mutation accumulation in diverse mutational mechanisms.

APOBEC deamination of cytosine to uracil (C>U) is thought to initiate mutations of signatures 2 and 13 (refs 11, 23, 24, 25). Knowledge of which of the Watson–Crick base pair is targeted by the APOBEC enzyme enables insight into the preferred replicative strand and indicates that APOBEC-related mutagenesis occurs at a higher rate on the p-to-q lagging replicative strand. APOBECs require single-stranded DNA as a substrate for cytosine deamination^26^,^27^ and thus the replication process itself could provide physiological opportunities for mutagenesis. Why APOBEC mutagenesis favours the lagging strand is unclear but could be due to differential availability of single-stranded DNA between leading and lagging strands for APOBEC deamination.

### Subtype heterogeneity in breast cancer and mutation distribution

A variety of breast cancer subtypes exist and these have been historically classified according to transcriptomic profiles. We sought to understand whether typical classifications such as oestrogen receptor subtype (positive or negative) or putative cell-of-origin (basal of luminal A/B) showed differences in the behaviour of mutational signatures, as this could confound the interpretation of our analyses. We found that mutational signature relationships to replication time (Supplementary Fig. 1g–k) and to replication strand (Supplementary Fig. 2e–i) were highly similar regardless of whether breast cancers were oestrogen receptor positive or negative or whether they were basal or luminal. Therefore, heterogeneity of the main breast cancer subtypes does not appear to influence the distribution of mutation signatures, suggesting that mutational processes behave relatively similarly in cells from the same tissue. It remains to be seen, however, whether mutational signatures will differ in their distributions when cancers of different tissue types are contrasted to one another.

### Processive mutagenesis

Previous work^28^ showed that APOBEC-related signatures 2 and 13 demonstrated strand-coordinated mutagenesis where pairs of adjacent mutations of the same reference allele were observed on the same strand more frequently than expected^28^. The underlying reason for this observation is unknown. Here we extend the strand-coordination analysis to identify long stretches of 10 or more successive mutations occurring on the same DNA strand (that is, successive mutations may be C>T…C>T…C>T or G>A…G>A…G>A but not C>T…G>A…C>T)^28^ for all mutation signatures. We found that long processive groups were features of signatures 2, 13, 6, 26 and 17 and were particularly over-represented in signature 13. 157 such processive groups were identified in 27 breast cancers, 76% of these from signature 13, which also had the longest processive group containing 19 point mutations (Fig. 2c, Supplementary Table 2).

The longest processive stretch of mutations covered ∼1.1  Mb although most processive stretches were tens to hundreds of kilobases in length (Supplementary Table 3). This suggests that mutational processes generating processive mutations can do so for remarkably long stretches, perhaps suggesting that long stretches of single-stranded DNA exist in cells and/or that individual proteins track one of the two DNA strands over long distances.

### Transcriptional strand biases

Base substitutions falling within the footprint of a gene, corresponding to ∼40% of the human genome, were classified according to whether they were on the ‘transcribed' strand (the non-coding strand), which forms the template for the primary mRNA transcript, or the ‘non-transcribed' strand^11^,^29^,^30^ (Fig. 2a,b, Supplementary Fig. 3).

Base-substitution signatures of breast cancer showed variation in transcriptional strand asymmetries. Signatures 2, 3, 5, 8, 13, 18 and 26 showed some transcriptional strand bias (*P* value <0.01 and strand imbalance up to 10.7%), while signatures 1, 6, 17, 20 and 30 showed no asymmetrybetween transcriptional strands (*P*-value >0.01).

While transcriptional strand bias can result from the involvement of transcription-coupled nucleotide excision repair that often acts on large, DNA distorting adducts^29^, the biases detected here in breast tissue are novel, and not easily ascribed to known double-helix distorting agents. These observations imply that other currently undefined transcription-coupled DNA damage and/or repair processes may be at play^31^,^32^,^33^ in breast tissue. The results also suggest that DNA replication has overall a stronger effect on the mutational landscape than transcription (Fig. 2a,b, Supplementary Table 1).

### Mutation signatures and nucleosome occupancy

**Figure 3:**
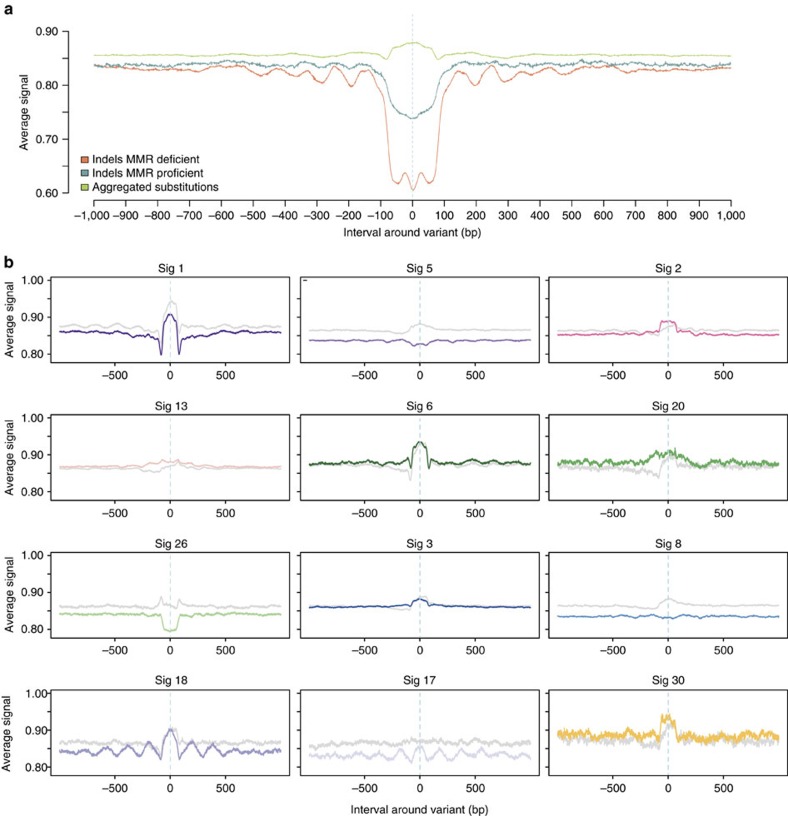
Relationship between mutational signatures and nucleosome occupancy. The distribution of the signal of nucleosome density (*y* axis) is shown in a 2 kb window centred on each mutation (position 0 on the *x* axis), for each signature. The averaged signal was calculated as the total amount of signal observed at each point divided by total number of mutations contributing to that signal. (**a**) Nucleosome density for aggregated substitutions (green), and for deletions observed in MMR-proficient (blue) and MMR-deficient (orange) samples. (**b**) Nucleosome density for the twelve base substitution signatures (note the degree of variation between substitution signatures relative to aggregated substitutions in **a**). The grey line shows the distribution predicted by simulations if mutations from each signature were randomly distributed. The analysis reveals that most of the observed distributions showed similar trends to those expected from simulations, apart from signatures 17, 18 and 26 and to a lesser extent signatures 5 and 8.

Finally, we examined how mutations due to different mutational signatures were distributed relative to nucleosome positions (Fig. 3, Supplementary Fig. 4). Nucleosomes consist of an octamer of histone proteins wrapped by ∼147 bp of ‘core DNA' and are separated from the next nucleosome by ∼60–80 bp of ‘linker DNA' sequence^18^. Reference regions indicating stable nucleosome occupancy were defined based on MNase experiments performed on an ENCODE line, K562 (refs 4, 34). Variant-to-nucleosome distances were calculated for each variant in each signature (Fig. 3a,b). A randomization was performed correcting for systematic variation in AT/CG content of mutational signatures and of core and linker DNA regions, and the distribution of observed mutations (Fig. 3b, coloured lines) was compared with that of the randomization (Fig. 3b, grey lines) for each signature.

Signature 17 and 18 mutations demonstrated enrichment at nucleosome core DNA sequences and showed a marked periodicity at ∼200–220 bp intervals, the approximate internucleosome distance^18^,^35^, contrasting with their predicted distributions through simulations and distinctly different when compared with all other signatures. By contrast, signature 26 mutations, associated with MMR deficiency, were more frequent at linker DNA sequences distant from nucleosomes^6^,^36^ (Fig. 3b). We also observed that repeat-mediated deletions, particularly those from MMR-deficient cancers were enriched at linker sequences (Fig. 3a) in keeping with previous reports^37^. Thus MMR deficiency increases the likelihood of indels as well as base substitutions occurring between nucleosomes. Intriguingly, it appears to do so in only one of the three distinct MMR-associated substitution signatures. Base-substitution signatures 1, 2, 3, 5, 6, 8, 13, 20 and 30 did not exhibit a different distribution to that expected from the randomization experiment suggesting that nucleosome positioning does not influence their underlying mutational processes.

## Discussion

This signature-based analysis is a novel way of visualizing *in vivo* mutagenesis providing a powerful means of revealing the natural experiments that occur in human cells. We find that different somatic mutational signatures demonstrate distinct partialities in replicative and transcriptional strands have variable profiles across replication time and are differentially influenced by physical genomic attributes such as nucleosome positioning (Table 1). The observed profiles are out-of-keeping with the profiles expected through randomization experiments that correct for per-sample mutation burden, AT/GC content of each signature and AT/GC content of the genome.

Reference coordinates for replication-related analyses were generated from Repli-Seq data for the cell line, MCF-7. Of publicly available Repli-Seq data sets, this ductal breast carcinoma cell line was selected because it was most closely related to the breast cancers in terms of tissue-of-origin. A large proportion of the earliest (average 59.8%, range 51.7–69%) and latest (average 77.9%, range 52.6–85%) replication domains are shared between MCF-7 and all other ENCODE cell lines (Supplementary Fig. 5), and analytical comparisons suggest that strong biological signals such as the replicative strand bias for signatures 2 and 13, remains convincingly consistent with only minor variation between cell lines (Supplementary Fig. 5, Supplementary Table 4). Thus, although a cell line will never be perfectly representative of what occurs *in vivo* in any cancer, MCF-7 appears to be a reasonable proxy for generating reference coordinates for assessing mutational distribution of breast cancers.

The signature-based analysis enables us to distinguish between similar mutational processes and provides mechanistic insights. Here we show that replication may be a source of single-stranded DNA over relatively long genomic distances, providing the potential for APOBEC-related deamination damage that initiates signatures 2 and 13. However, mathematical extractions differentiate these signatures and suggest that signature 2 is composed predominantly of C>T transitions, while signature 13 comprises mainly C>G transversions^11^,^14^. These differences have been hypothesized to be due to the subsequent reparative step^11^,^13^: it was postulated that C>G signature 13 transversions were fixed via uracil-N-glycosylase (UNG)- and REV1-polymerase (REV1)-dependent mechanism in base excision repair, while C>T signature 2 transitions were formed by replicative polymerases exerting the ‘A' rule (the preferential insertion of adenine opposite non-informative templates such as abasic sites or uracils^38^).

**Figure 4:**
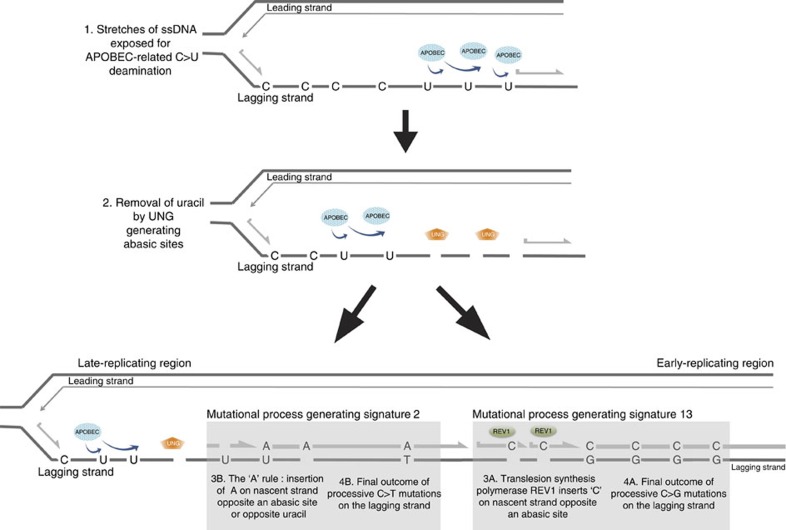
A replication-related model of mutagenesis for putative APOBEC-related signatures 2 and 13. 1. During replication, transient moments of increased availability of single-stranded DNA (ssDNA) (for example, uncoupling between leading and lagging replicative strands or delays in elongation of the nascent lagging strand by Okazaki fragments) could occur, exposing ssDNA for APOBEC deamination, potentially for long genomic tracts. 2. Uracil-N-glycosylase (UNG) acts to remove undesirable uracils leaving a trail of abasic sites in its wake. Divergence of mutational processes occurs from this point. 3A Earlier in replication, error-prone translesion polymerases such as REV1 have been postulated to insert cytosines opposite abasic sites to avoid detrimental replication fork stalling or collapse. 4A The final outcome is stretches of successive C>G transversions at a TpC sequence context characteristic of signature 13. 3B Alternatively, uracils and abasic sites that are not fixed via REV1, undergo contingency processing, for example, the ‘A' rule. 4B The final outcome is of C>T mutations at a TpC sequence context.

Here analyses relating to replication dynamics show that first, signatures 13 and 2 have distinct replication strand biases, second, signature 13 has consistently longer processive groups than signature 2 and third, signature 13 mutations are more frequent early in replication than signature 2. We postulate that UNG/REV1-dependent uracil processing generating signature 13 mutations occurs earlier in replication, thus has more time and is more likely to process successive uracils. By contrast, the reparative process that generates signature 2 mutations acts on remaining incompletely processed uracils and/or abasic sites including those that are left at the end of the replication cycle, leading to the observed distribution of higher frequencies late in replication. Thus, we suggest a replication-related model of APOBEC mutagenesis: although the replication process itself springs forth opportunities for APOBEC-related DNA damage, it is possible that the variation in repair across replication time results in these disparate signatures (Fig. 4).

Signatures 6, 20, 26 and an excess of repeat-mediated indels are all associated with defective MMR^23^. They are rare signatures (only 1.7%) in breast cancers, often found together in the same breast tumours and are overwhelming when they occur. However, they exhibit differing relationships with respect to replication time and direction, transcription and nucleosome occupancy (Table 1). Intriguingly, only one of the three MMR-related substitution signatures exhibits a distinctive distribution relative to nucleosome occupancy, an observation that would not have been appreciable without this signature-based approach applied to WGS data.

Three of six rearrangement signatures (RS1, RS3 and RS5) are associated with defects in HR and are enriched early in replication. Microhomology-mediated indels and substitution signatures 3 and 8 are also associated with HR defects but are enriched late in replication. These signatures are often found together (albeit in differing quantities) in individual patients^15^ and likely represent compensatory methods of double-strand break (DSB) repair in the face of defective HR.

Perhaps, back-up recombination-based repair pathways^39^,^40^,^41^,^42^ are more likely to be invoked early in replication because DSBs encountered early in S-phase are poorly tolerated^42^,^43^,^44^,^45^. In contrast, microhomology-mediated deletions represent the outcome of DSB resolution through microhomology-mediated end joining mechanisms, reflecting a contingency route for DSBs that have not been repaired successfully by recombination strategies^44^,^45^ earlier in replication. Likewise, base substitution signatures 3 and 8 could similarly represent the outcome of back-up error-prone translesion synthesis activity in HR-deficient cancers^46^. Regardless, these different compensatory signatures exhibit distinctive behaviours across replication time, painting the physiology of HR deficiency as complex mutagenesis that has an instantly recognizable whole-genome profile with potential clinical relevance^15^.

To the best of our knowledge, we provide the first systematic signature-based characterization of the genomic distribution of all classes of somatic mutations in human cancer. The power provided by large numbers of WGSs of a single cancer type affords a higher resolution perspective on the topography of biological processes underlying mutagenesis in breast tissue. We emphasized how detailed analyses help showcase the mechanistic contribution of replication dynamics to specific mutational processes (for example, APOBEC-related signatures 2 and 13). We also highlighted how multiple forms of DNA repair have an impact on mutation distribution leaving complex but distinctive global genomic profiles. Finally, the signature-based genomic variation seen here drives home a fundamental point regarding genomic analyses forthwith: statistical models involving mutability cannot assume uniform genomic mutation rates and must consider signature-dependent variation as a factor in all future analyses.

## Methods

### Data set

All short-read sequencing data were aligned on the GRCh37/hg19 assembly^15^ and somatic substitutions, indels and rearrangements called and curated as previously described^15^. High-quality mutation calls were parsed through non-negative matrix factorization^11^,^12^,^14^,^47^ to extract mutation signatures. In all, twelve base substitution signatures and six rearrangement signatures were identified^15^. Indels were classified according to the properties of the breakpoint junctions into two signatures. Insights into mechanisms generating deletions are more certain than that of insertions, thus our analyses were restricted to deletions.

The non-negative matrix factorization-based mutational signatures analysis revealed the substitution signatures of 12 mutational processes operative in breast cancer^15^. Furthermore, the analysis provided the number of somatic mutations assigned to each of these 12 signatures in each of the examined breast cancers (exposures). Using the patterns of the extracted mutational signatures and their contributions in each sample, we were able to assign an *a posteriori* probability for any individual substitution to be generated by any of the 12 mutational signatures in a given sample. The posterior probability for a given substitution with a trinucleotide context, *k*, to be generated by the mutational signature *n* in the sample *g*, (^*k*^_*ng*_), was computed as the exposure of this sample to the signature *n* (*e*^*n*^_*g*_), multiplied by the probability of the signature *n* to generate this particular mutation with trinucleotide context *k* (*p*^*k*^_*n*_). The *a posteriori* probabilities were then normalized to sum to 1 by using the number of mutations observed in the sample 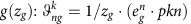.

Different methodologies were considered to associate the substitutions with the mutational processes that generated them: (i) maximum likelihood (the signature associated with each mutation was the one having the highest probability), (ii) maximum likelihood with probability threshold (same of maximum likelihood but here signatures with an a posteriori probability lower or equal to 0.5 were filtered out), (iii) belief propagation (the a posteriori probabilities ϑ^*k*^_*ng*_ were propagated in the downstream analyses).

We used the maximum likelihood approach to perform the analyses described in the main manuscript. This choice was motivated by the fact that this approach could be consistently applied to all downstream analyses and could be used to perform statistical tests. For example, belief propagation could not be used for the analysis of processive groups and it was not suitable for statistical tests requiring integer values. In addition, the thresholding method tended to result in reduced power. Regardless, the strong biological signals from analyses of particular signatures such as signatures 2 and 13 were robust and reproducible across all three approaches (to compare the different methodologies, please see results from the thresholding and belief propagation approach in Supplementary Fig. 6).

### Replication analyses

Reference coordinates for replication landmarks were inferred from Repli-seq data obtained from the ENCODE project^48^ (https://www.encodeproject.org/). Cell lines were first isolated into six cell cycle fractions of newly replicated DNA (G1/G1b, S1, S2, S3, S4 and G2) and each fraction was sequenced. To visualize genome-wide replication patterns as a continuous function, percentage-normalization of sequencing tags was followed by a wavelet-smoothed transformation.

The majority of origins do not fire as a part of a clear, deterministic programme, instead origin firing occurs both individually and as clusters^49^,^50^,^51^. Replication domains were defined using Repli-seq signal: peaks (local maxima) in the smoothened profile correspond to replication initiation zones, while valleys (local minima) correspond to replication termination zones^48^. Replication time domains were modelled on conservatively defined transition zones in DNA replication time data. Repli-seq data were split into deciles with each segment containing exactly 10% of the observed signal. AT/CG content of the deciles were variable (Supplementary Fig. 1a), and the genome-wide distribution of the deciles was heterogeneous (Supplementary Fig. 1b).

All analyses related to replication time domains were corrected for genomic size. In particular, in each decile a mutation density was computed as the total mutation count in each decile divided by the number of attributable bases (excluding ‘N's) contained in the relevant decile. In order for gradients to be comparable between signatures (given the variation in mutation rate between signatures), counts were then normalized to between 0 and 1. Results of analyses with absolute counts can also be found in Supplementary Fig. 1c–e.

Finite difference approximations of second and first derivatives were used to identify Repli-seq signal local maxima (*f ′′(x)*<0) and local minima (*f ′′(x)*>0) corresponding to potential origin firing sites, and then to distinguish between leading (*f ′(x)<*0) and lagging (*f ′(x)*>0) strand, respectively (Supplementary Fig. 2a–b). Derivative functions were defined in agreement with p and q arm chromosome orientation. We named the replication strand as p2q leading and p2q lagging. To remain conservative in downstream assignments^52^,^53^, we removed the last 25 kb of the latest zones of the replicating domains. We focused on long transitions between early and late replicative domains, discarding ambiguous mini-peaks or valleys that were <10 kb in length. It was possible to assign replication domains in 2,414,428,423 bp of the genome. The median length of assigned replication strand was 136,001 bp and the mean length was 196,907 bp, safely and conservatively within the limits described by recent alternative methods, including DNA combing^54^. Derived p2q leading and p2q lagging strands were comparable in genomic footprint, AT/CG content and in amount of transcribed/non-transcribed regions (Supplementary Fig. 2c–d).

To investigate asymmetry relative to replication strands, all base substitutions were first described in the pyrimidine context and then orientated with respect to the relevant strand.

### Choice of reference cell line for mutational distribution

Replication time-related coordinates for the main analyses reported in the manuscript were generated from MCF-7 cell line. This choice was motivated by the fact that this is a ductal breast carcinoma cell line and most closely represented our data set of breast cancers. Note that across the 14 cell lines available from ENCODE, on average 59.8% of the earliest replication time domain is shared between MCF-7 and each of the other cell lines (range 51.7–69%), and average 77.9% of the latest replication time domain (range 52.6–85%; Supplementary Fig. 5a). In other words, large swathes of the earliest and latest replication time domains are identical between MCF-7 and other cell lines.

To further contrast the cell lines to identify the most appropriate source of reference coordinates for our analyses, we analysed the mutation density trend across the cell cycle for all cell lines where data were available from ENCODE. Cell lines showing a consistent increase of aggregated mutation density going from early to late replicative regions, should be preferred over the cell lines that exhibited a random trend. For each cell line, we extracted replication time deciles and counted the number of mutations falling in each of these domains. These counts were then corrected for the genomic size of each domain. In this way, we obtained the mutation density *m*^*i*^_*j*_ for each decile (*i* represents the *i*-th cell line, and *j=*1,2,..,10 the decile with 1 and 10 being the earliest and the latest decile, respectively). The mutation densities were ordered across replication time (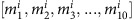) to capture the overall trend of mutation accumulating across the cell cycle. Pearson's correlations were separately applied to assess the relationship between the distribution of mutations across replication time domains (expecting an increasing trend progressing through to late replication). The Pearson's test showed low *P* values for strong correlations across replication time. On the contrary, less-significant *P* values were observed for distributions that were poorly correlated and showed more randomly distributed mutations across replication time domains. Results of this comparison are showed in Supplementary Fig. 1f.

### Transcriptional strand characterization

The nucleotide sequence of the primary mRNA transcript is identical to the sense/non-template/non-transcribed strand except that U replaces T, and is complementary to that of the anti-sense/template/transcribed strand (Supplementary Fig. 3a).

All mutations were called on the + strand of the reference genome, were placed into the ‘pyrimidine' context and noted if so. Transcriptional strand was assigned for each pyrimidine-based mutation (Supplementary Fig. 3b for explanation of orientation). Regions of the genome with protein coding genes were used to assign transcriptional strands. On the total of 20,305 protein coding genes, 10,301 (677,912,252 bp) were on the + strand and 10,004 (646,112,188 bp) were on the − strand, respectively.

### Computing replication and transcription strand ratios

All base substitution mutations were described in the pyrimidine context and orientated with respect to the replication and transcription strand (for example, an A>C observed on the p2q leading strand was counted as a T>G on the lagging). Given the broadly random orientation of both transcriptional direction of genes in the genome and replication strand (Supplementary Fig. 2c–d), our null hypothesis is that all the signatures would have a 50:50 distribution with respect to transcriptional or replicative strands. To ensure this hypothesis is robust to other features in the genome effecting mutation rates, such as local base composition, we randomized the position of the observed mutations keeping the local triplet context (see statistical analysis section below for more details about the approach used to generate the simulated data). The random simulations showed no bias towards either replication strand or transcription strand (all *P* values >0.05, binomial test). In contrast, many signatures showed striking bias either in replication strand or transcription strand with the deviating signatures showing strong statistical support (all *P* values <2e^−16^ binomial test; Fig. 1b and Supplementary Table 1).

Supplementary Figure 2e and 2f show the overall distribution of the ratios for the six mutation classes and for the 12 signatures, respectively, across all the 560 samples (each dot represents a sample), with summary plots in Supplementary Fig. 2g–h.

Note that the interpretation of transcriptional or replicative strand bias for six classes of base substitutions is restricted by the complexity of mutational mechanisms that contribute to each base substitution class (Fig. 2a,b). For example, C>T transitions exhibit lagging replicative strand bias (Supplementary Fig. 2g) but are components of signatures 1 (due to deamination of methylated cytosines), 2 (APOBEC related), 6, 20, 26 (MMR deficiency) and 30 (unknown aetiology). Hence, our analyses concentrate on exploring extracted base substitution signatures (Supplementary Fig. 2h) instead of the six classes of base substitutions (Fig. 2a, Supplementary Fig. 2g), but are provided for information.

### Processivity

Processive groups were defined separately for each sample. Kataegis mutations were excluded from these analyses first as they have been previously highlighted to demonstrate strand-coordinated mutagenesis thus inclusion of kataegis would produce a biased signal.

Adjacent somatic mutations were considered to be part of the same processive group if (i) they were associated with the same signature and (ii) they had the identical reference allele (complementary mutations were not considered processive e.g., A>G, T>C, A>G=non-processive while A>G, A>G, A>G or T>C, T>C, T>C=processive).

The average mutation density for processive groups for each signature is provided in Supplementary Table 2.

A total of 426,066 processive groups were identified. We characterized each group by using the number of substitutions involved in each of them (processive group length). Group length ranged from 2 to 19 (Supplementary Table 2).

Results shown in Fig. 1c were generated by counting the number of groups having the specified length for each mutation signature. For visualization purposes, the absolute counts were log10 transformed. We used a set of simulated mutations to understand whether the observed processive behaviour was the consequence of the idiosyncrasies of individual samples (that is, the possibility of observing long processive groups in samples containing many mutations belonging to one signature may be higher than samples having fewer mutations or having mutations belonging to several different signatures).

We generated a null distribution by using 100 random data sets that took the number and type of mutations relevant to each signature into consideration (more details on the approach used to generate the random simulations can be found in Relationships with nucleosome occupancy Section. To assess the probability of observing groups of a particular length, we compared the observed data to the null distribution. Let *p*^*n*^_*ij*_ be the number of processive groups of length *i* observed in the *j*th random dataset and associated with the signature *n*. We can compute the number of processive groups of length *i* observed across all the 100 simulated datasets for the signature *n* as 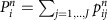, (*J*=1, …, 100), and we can assess the probability to observe a processive group of length *L* for the signature *n* as: 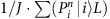. Bonferroni's correction was used to adjust for multiple testing.

For each signature the replication strand ratio was computed by summing the mutations over all the groups having the specified length. Two-tailed binomial test was used separately on each group to assess the significance of the imbalance between leading and lagging strand, and 0.05 was used as the level of significance in this analysis. Note that groups with less than six mutations did not contain enough observations to obtain a significant *P* value from the two-tailed binomial test.

Intriguingly, we also observed that all or nearly all mutations were on the same replicative strand within individual processive groups for signature 13. Indeed, lagging strand bias was ∼fourfold stronger for processive mutations than those that were not processive. The data implies that for signature 13, replication is not simply a source of single-stranded DNA, it permits processive deamination for exceptionally long genomic distances.

### Relationships with nucleosome occupancy

Micrococcal nuclease sequencing (MNase-seq) data for the K562 cell line was obtained from the ENCODE project^48^. Please see http://genome.ucsc.edu/cgi-bin/hgTrackUi?db=hg19&g=wgEncodeSydhNsome for details of experiments and generation of nucleosome density signal maps. Although K562 is not a breast cancer cell line, our choice to use it for our analysis was motivated by several factors: it has been used in other research laboratories for similar analyses^55^, it is one of the two main reference lines archived in ENCODE with clear cell culture protocols for these experiments and it is the only cancer cell line available.

To assess the relationships between signatures and nucleosome occupancy, we created a window of 2 kb centred around each mutation (within a signature), and obtained the nucleosome density signal observed within the 2 Kb window. We calculated the SUM of the signal observed across the window for all the mutations within a signature, and the number of mutations (COUNT) contributing to the signature. The average signal (*y* axis) is the SUM/COUNT for every position within the 2 kb window (Supplementary Fig. 4a–b). The nucleosome density signal distribution for K562 MNase data encompasses 575,649,742 loci, where the signal is a smoothed version of the total number of reads. The MNase signal has a skewed distribution with mode that lies in the interval 0.85–0.9, hence the averaged signal (accounting for all the mutations) for each signature lies in the region of 0.85–0.9.

Note that every mutation that contributes towards a given signature is at a different genomic location and there are many thousands, or even tens or hundreds of thousands of mutations per signature. If mutations in a given signature bore no relationship to the position of nucleosomes, then when aggregated across thousands of mutations per signature, a flat line would be seen. However, if mutations in a particular signature were more frequent at core sequences, there would be a peak of nucleosome signal where the mutation is centred. If mutations were more frequent at linker sequences, there would be a trough.

### Other computational and statistical analyses

All statistical analyses were performed in R (version 3.0.2): Pearson's correlation was performed with cor.test, binomial test was performed with binomial test, Bonferroni correction was performed by using p.adjust. Multivariate normal mixtures were computed by using normalmixEM function available as part of the R/CRAN mixtool package, an initial mixing proportion of 0.5 was used to compute three components (parameter *lambda* and *k* of the function, respectively). corrplot R package was used to generate Fig. 1c and Supplementary Fig. 1b and 5a. Data in bigWig format were preprocessed by using bigWigToBedGraph script. Perl EnsEMBL API (version 73) was used to extract the genome features of interest. bedtools (version 2.16.2) was used to identify intersection and union among genomic features, and to manipulate BED files. samtools (version 0.1.18) was used to extract subsequences from fasta files.

Random mutations were generated in agreement with their flanking sequence context defined by the neighbouring bases immediately 5′ and 3′ to the mutated base and by the mutated base itself. We generated random simulations of the dataset obtained from the 560 breast cancers. We imposed the following constraints: (i) mutation class (ii) flanking sequence context (iii) overall mutation burden (iv) contribution of each mutation signature to each sample.

To perform simulations of processive data, mutations for each signature were shuffled separately for each sample. Shuffled samples were then used to compute the processive groups. For each signature 100 simulations were used to compute the null distribution associated with the expected processive length associated.

For each observed rearrangement we simulated both breakpoint junctions, and we kept the signature, and type (that is, translocation, inversion, deletion and tandem duplication) observed in the real dataset. Given a rearrangement we randomly picked the first breakpoint from one of the replication time deciles, then (i) if the rearrangement was not a translocation then we randomly pick the second breakpoint on the same chromosome of the first one (ii) if the rearrangement was a translocation we randomly picked also the second breakpoint without any constraint.

Functional genomics experiments based on next-generation sequencing, such as Repli-seq and MNase-seq, often produce artefactual signals in certain regions of the genome. To filter out artefact-ridden regions that tend to show artificially high/low signals, we excluded from our analyses ENCODE blacklisted genomic regions (human, hg19/GRCh37):

http://hgdownload.cse.ucsc.edu/goldenpath/hg19/encodeDCC/wgEncodeMapability/wgEncodeDacMapabilityConsensusExcludable.bed.gz.

## Additional information

**How to cite this article:** Morganella, S. *et al*. The topography of mutational processes in breast cancer genomes. *Nat. Commun.* 7:11383 doi: 10.1038/ncomms11383 (2016).

## Supplementary Material

Supplementary Information Supplementary Figures 1-6 and Supplementary Tables 1-4.

## Figures and Tables

**Table 1:** Summary of relationships between each mutational signature and various genomic features.

Mutational signature	Mutation type	Predominant features of signature	Associated mutational process	Transcriptional strand	Replicative strand	Replication time	Chromatin organization
1	Sub	C>T at CpG	Deamination of methyl-cytosine (age associated)		Some bias	Enriched late	
5	Sub	T>C	Uncertain (age associated)	Some bias	Some bias	Enriched late	Slight enrichment at linker
2	Sub	C>T at TpCpN	APOBEC related	Some bias	Strong lagging strand bias	Enriched late	
13	Sub	C>G at TpCpN	APOBEC related	Some bias	Strong lagging strand bias	Flat	
6	Sub	C>T (and C>A and T>C)	MMR deficient		Some bias	Flat	
20	Sub	C>A (and C>T and T>C)	MMR deficient		Some bias	Enriched late	
26	Sub	T>C	MMR deficient	Some bias	Strong bias	Enriched late	Enriched at linker
3	Sub		HR deficient	Some bias	Some bias	Enriched late	
8	Sub	C>A	amplified by HR deficiency?	Some bias		Enriched late	
18	Sub	C>A	Uncertain	Some bias	Some bias	Enriched late	Enriched at nucleosomes and periodic
17	Sub	T>G	Uncertain		Some bias	Enriched late	Enriched at nucleosomes and periodic
30	Sub	C>T	Uncertain			Flat	
RS1	Rearr	Large tandem duplications (>100 kb)	Uncertain type of HR deficiency?	NA	NA	Enriched early	
RS2	Rearr	Dispersed translocations		NA	NA	Enriched early	
RS3	Rearr	Small tandem duplications (<10 kb)	HR deficiency (BRCA1)	NA	NA	Enriched early	
RS4	Rearr	Clustered translocations		NA	NA	Enriched early	
RS5	Rearr	Deletions	HR deficient	NA	NA	Enriched early	
RS6	Rearr	Other clustered rearrangements		NA	NA	Enriched early	
Repeat-med	Indel	<3 bp indel at polynucleotide repeat tract	amplified when MMR deficient	NA	NA	Enriched late	Enriched at linker and periodic
Microhom	Indel	≥3 bp indel with microhomology at breakpoint junction	HR deficient	NA	NA	Enriched late	

HR, homologous recombination; indel, insertions/deletions; rearr, rearrangement; RS, rearrangement signature; sub, substitution.

The 20 mutational signatures are noted in the left most column. This is followed by information on mutation classes, features that predominantly characterize each signature and associated aetiologies, if known. Relationships relating to transcriptional strands, replication time and strands and chromatin organization are also noted.
