# Supplementary material for: The topography of mutational processes in breast cancer genomes
Source: Nat Commun. 2016 May 2;7:11383. doi: 10.1038/ncomms11383 (PMC5001788; doi:10.1038/ncomms11383)
Supplement: Supplementary Information — Supplementary Figures 1-6 and Supplementary Tables 1-4. [file ncomms11383-s1.pdf]

A

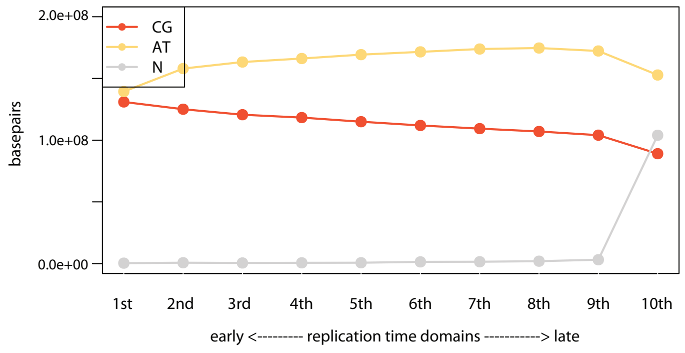

CG
AT
N
2.0e+08
1.0e+08
0.0e+00
basepairs
1st
2nd
3rd
4th
5th
6th
7th
8th
9th
10th
early <--------- replication time domains -----------> late

B

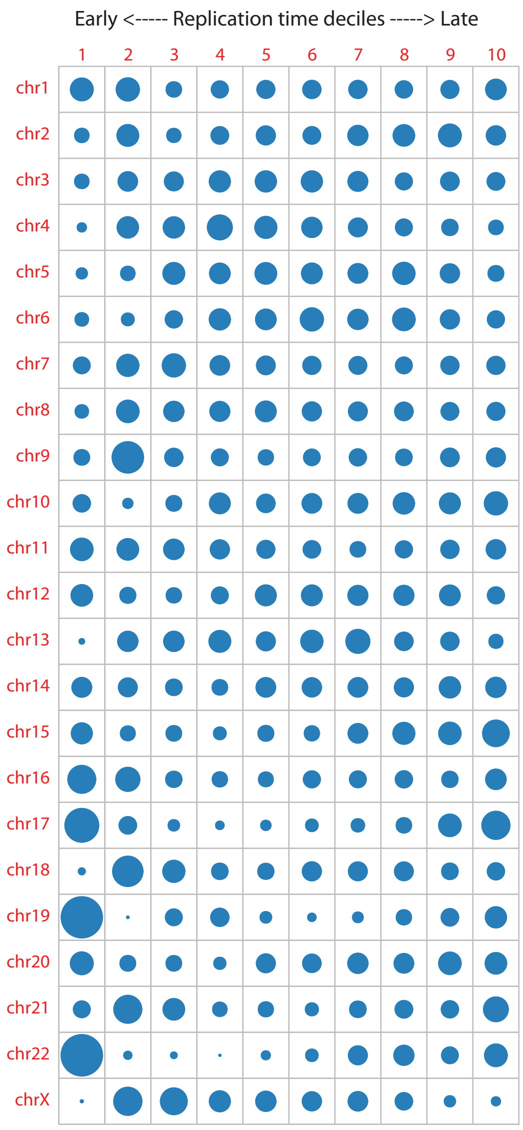

Early <----- Replication time deciles -----> Late
1
2
3
4
5
6
7
8
9
10
chr1
chr2
chr3
chr4
chr5
chr6
chr7
chr8
chr9
chr10
chr11
chr12
chr13
chr14
chr15
chr16
chr17
chr18
chr19
chr20
chr21
chr22
chrX

C

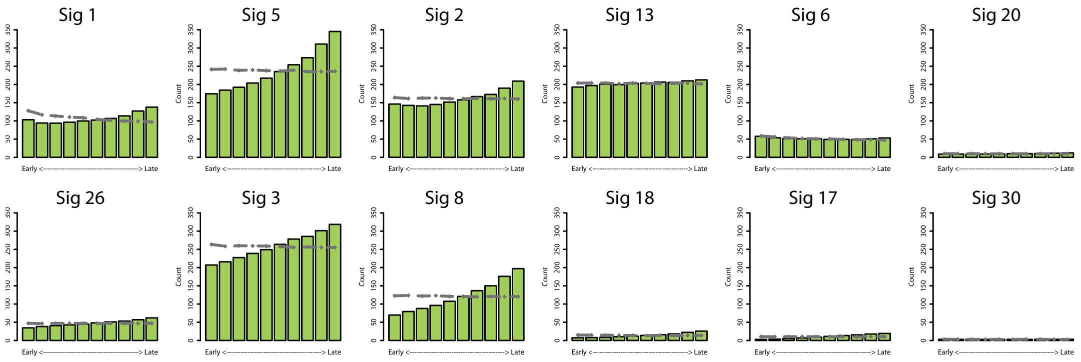
Sig 1
Sig 5
Sig 2
Sig 13
Sig 6
Sig 20
Sig 26
Sig 3
Sig 8
Sig 18
Sig 17
Sig 30
Count
Early <------------------------------> Late

D

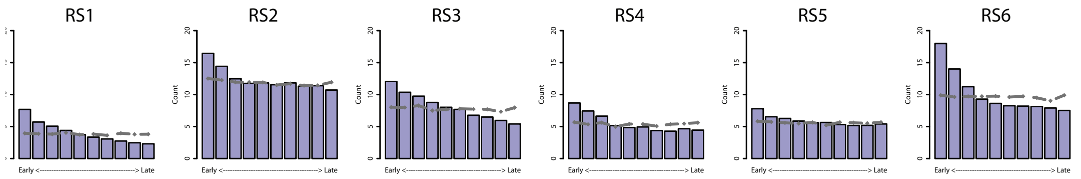
RS1
RS2
RS3
RS4
RS5
RS6
Count
Early <------------------------------> Late

E

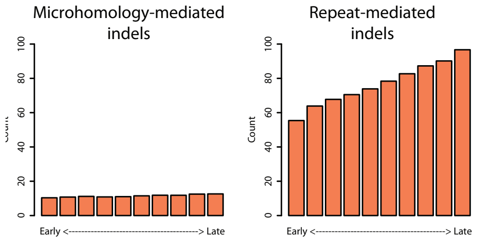
Microhomology-mediated indels
Repeat-mediated indels
Count
Early <------------------------------> Late

F

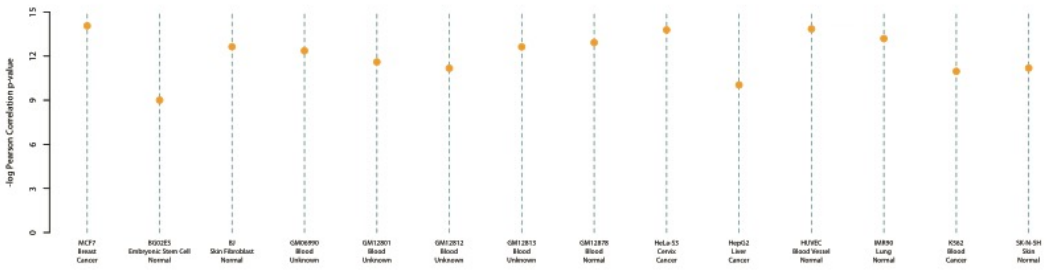
-log Pearson Correlation p-value
MCF7 Breast Cancer
BG02ES Embryonic Stem Cell Normal
BJ Skin Fibroblast Normal
GM06990 Blood Unknown
GM12801 Blood Unknown
GM12812 Blood Unknown
GM12813 Blood Unknown
GM12878 Blood Normal
HeLa-S3 Cervix Cancer
HepG2 Liver Cancer
HUVEC Blood Vessel Normal
IMR90 Lung Normal
K562 Blood Cancer
SK-N-SH Skin Normal

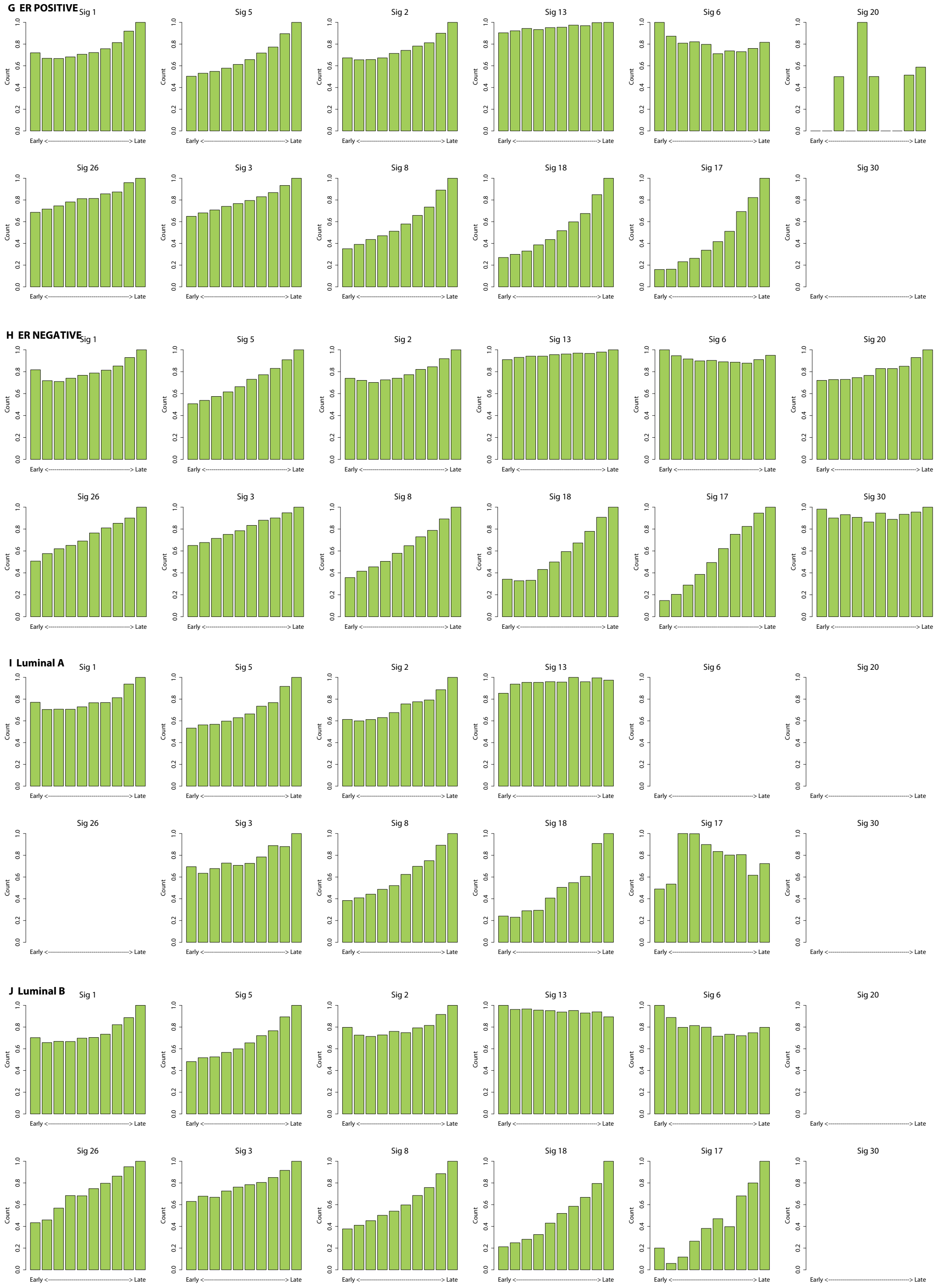
G ER POSITIVE
Sig 1
Sig 5
Sig 2
Sig 13
Sig 6
Sig 20
Sig 26
Sig 3
Sig 8
Sig 18
Sig 17
Sig 30
H ER NEGATIVE
Sig 1
Sig 5
Sig 2
Sig 13
Sig 6
Sig 20
Sig 26
Sig 3
Sig 8
Sig 18
Sig 17
Sig 30
I Luminal A
Sig 1
Sig 5
Sig 2
Sig 13
Sig 6
Sig 20
Sig 26
Sig 3
Sig 8
Sig 18
Sig 17
Sig 30
J Luminal B
Sig 1
Sig 5
Sig 2
Sig 13
Sig 6
Sig 20
Sig 26
Sig 3
Sig 8
Sig 18
Sig 17
Sig 30
Count
Early <------------------------------> Late

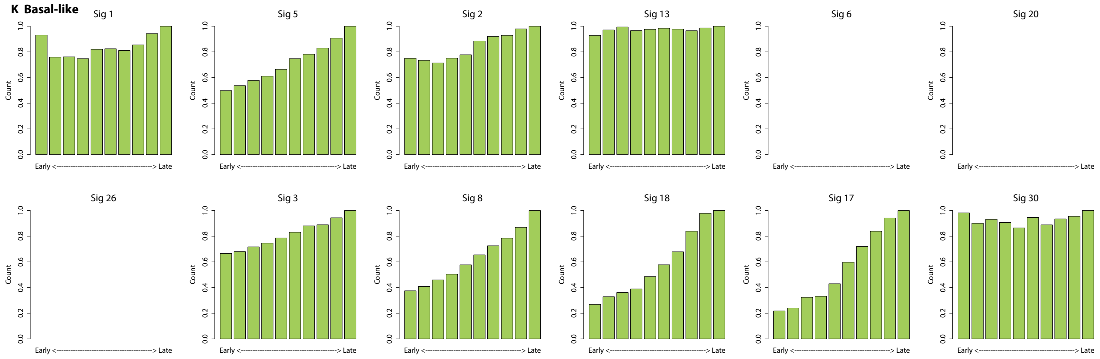

Supplementary Fig. 1 | Characterization of replication time domains. A, Distribution of CG/AT and N across the replication time deciles extracted from MCF-7 Repli-seq data. **B,** Overview of distribution of replication time domains across chromosomes. The size of each circle is proportional to the percentage of DNA in the chromosome (row) that is present in a specific decile (column). Absolute counts across the replication time deciles of **C,** mutations **D**, rearrangements and **E**, indels by signature. The dashed-diamond lines show the trend expected from the randomization experiments. **F,** Exploring correlations between replication time and somatic mutations across fourteen cell lines. Y-axis shows the –log of the *p*-value obtained from a Pearson's correlation test that estimates the relationship between aggregated base substitutions and replication timing domains for each cell line. **G-K,** Replication time analysis for the twelve base substitution signatures, where samples were grouped together by using ER status (ER positive 366 samples and ER negative 194 samples), and gene expression profiles LumA (101 samples), LumB (75 samples) and Basal (74 samples) respectively [see [15] for further details on complete cohort].

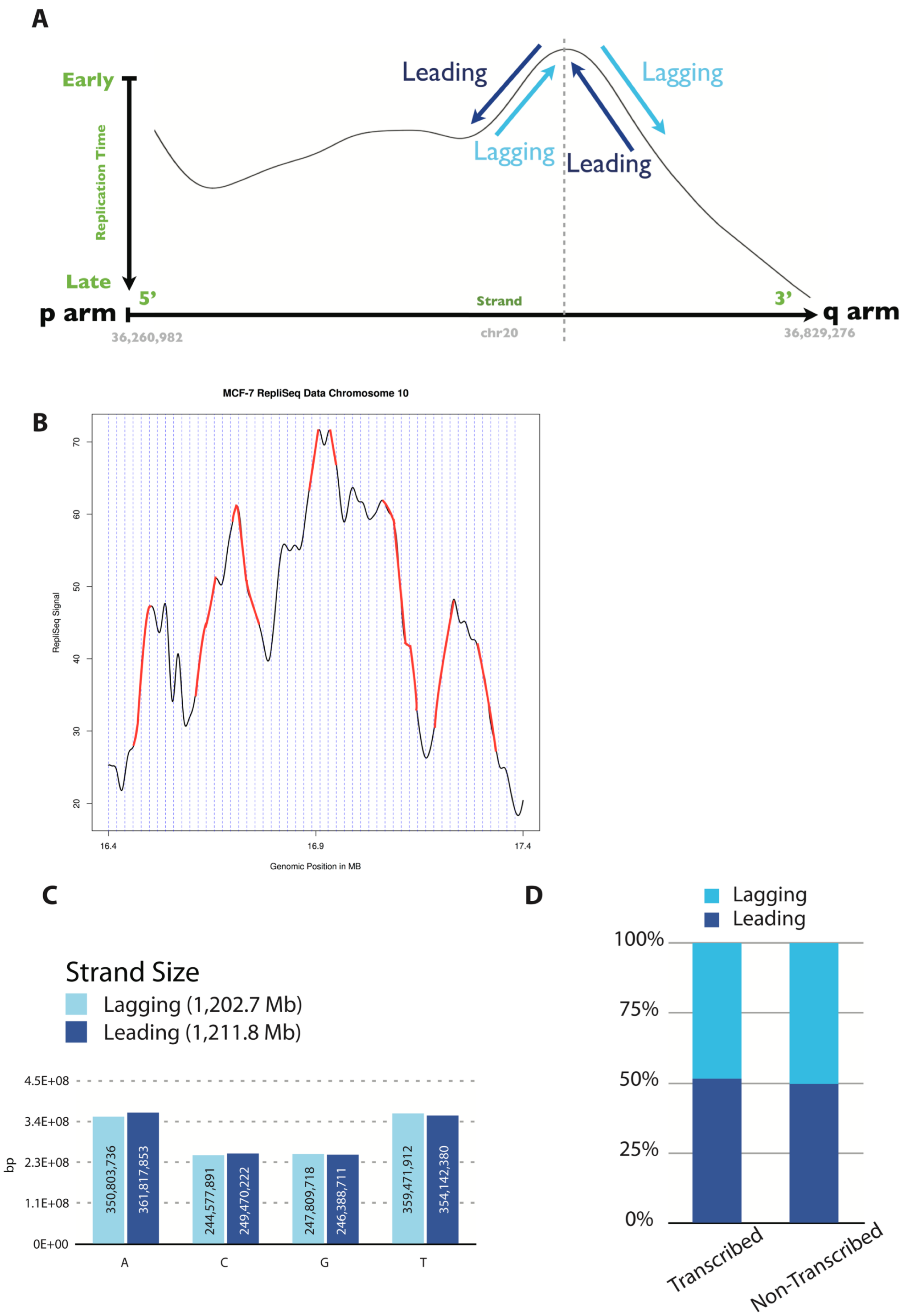
A
Early
Replication Time
Late
Leading
Lagging
Lagging
Leading
5'
3'
p arm
Strand
q arm
36,260,982
chr20
36,829,276
B
MCF-7 RepliSeq Data Chromosome 10
RepliSeq Signal
Genomic Position in MB
16.4
16.9
17.4
C
Strand Size
Lagging (1,202.7 Mb)
Leading (1,211.8 Mb)
bp
4.5E+08
3.4E+08
2.3E+08
1.1E+08
0E+00
350,803,736
361,817,853
244,577,891
249,470,222
247,809,718
246,388,711
359,471,912
354,142,380
A
C
G
T
D
Lagging
Leading
100%
75%
50%
25%
0%
Transcribed
Non-Transcribed

E

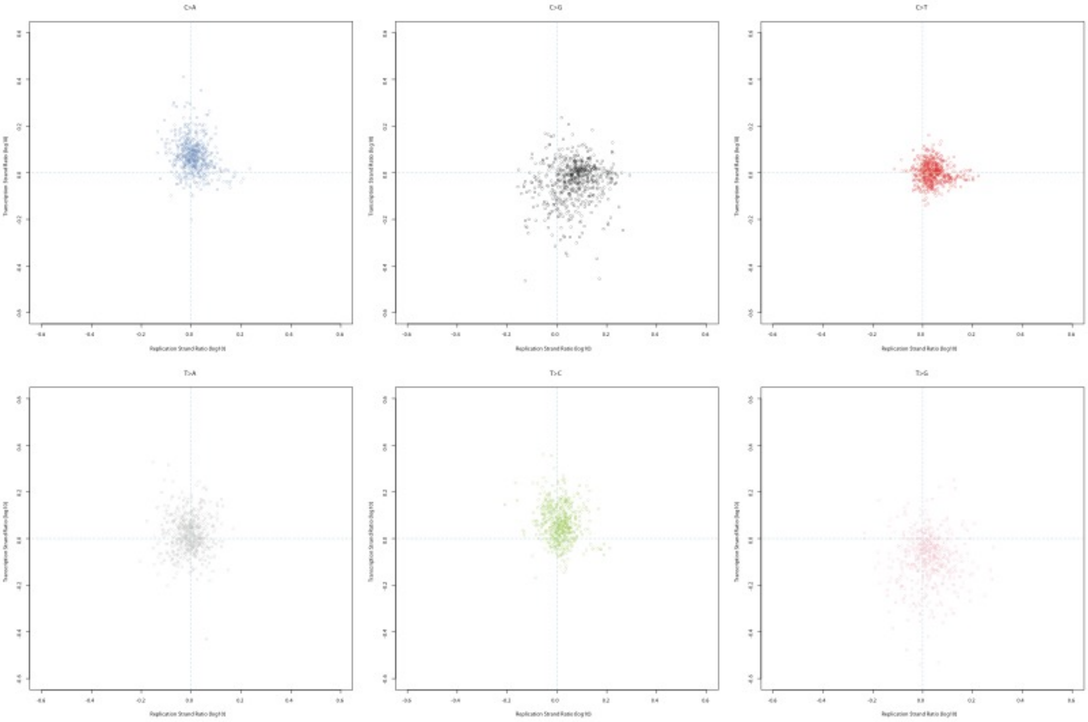
C>A
C>G
C>T
T>A
T>C
T>G
Replication Strand Ratio (log10)
Transcription Strand Ratio (log10)

F

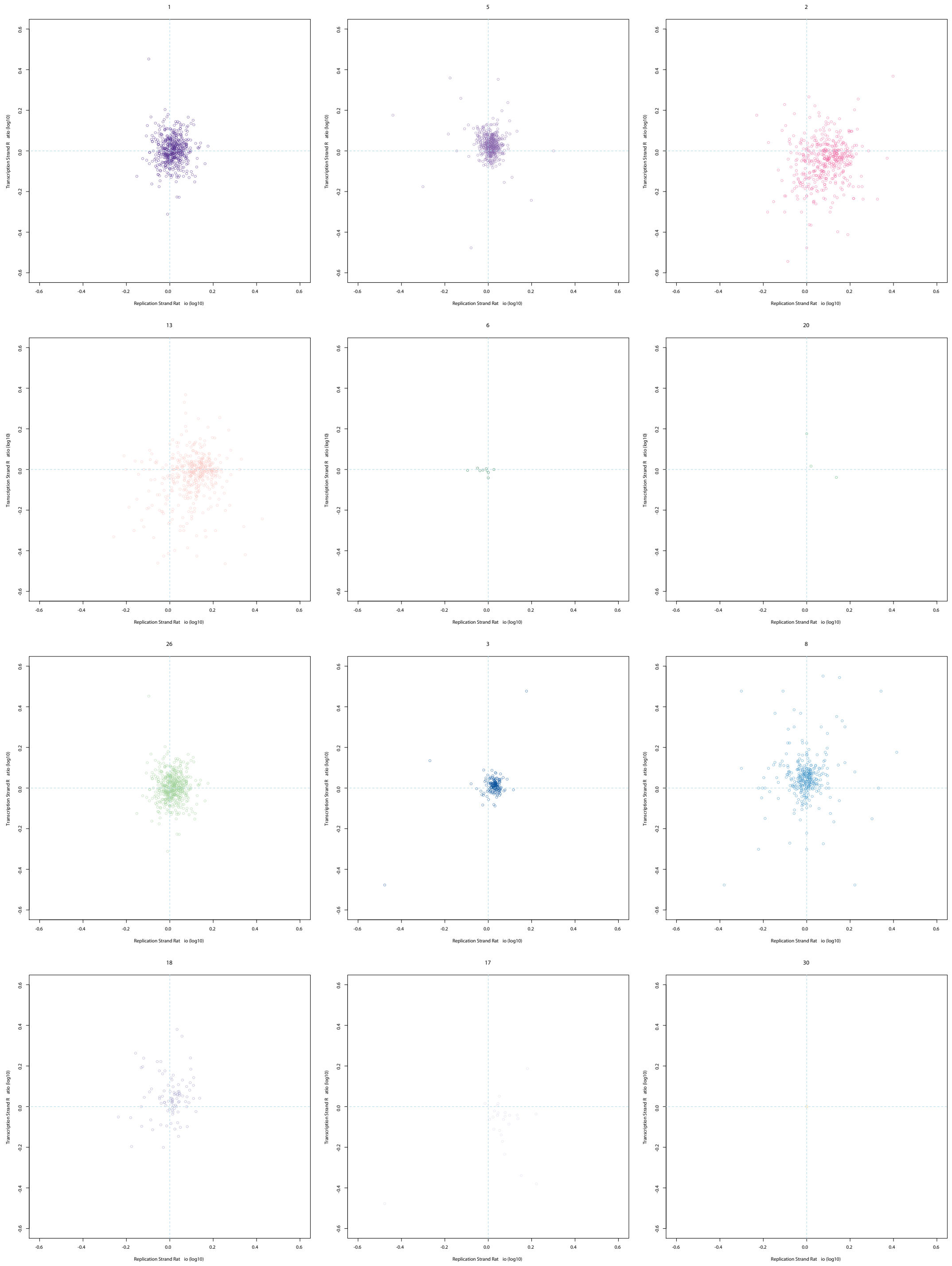
1
5
2
13
6
20
26
3
8
18
17
30
Replication Strand Rat io (log10)
Transcription Strand R atio (log10)
-0.6
-0.4
-0.2
0.0
0.2
0.4
0.6

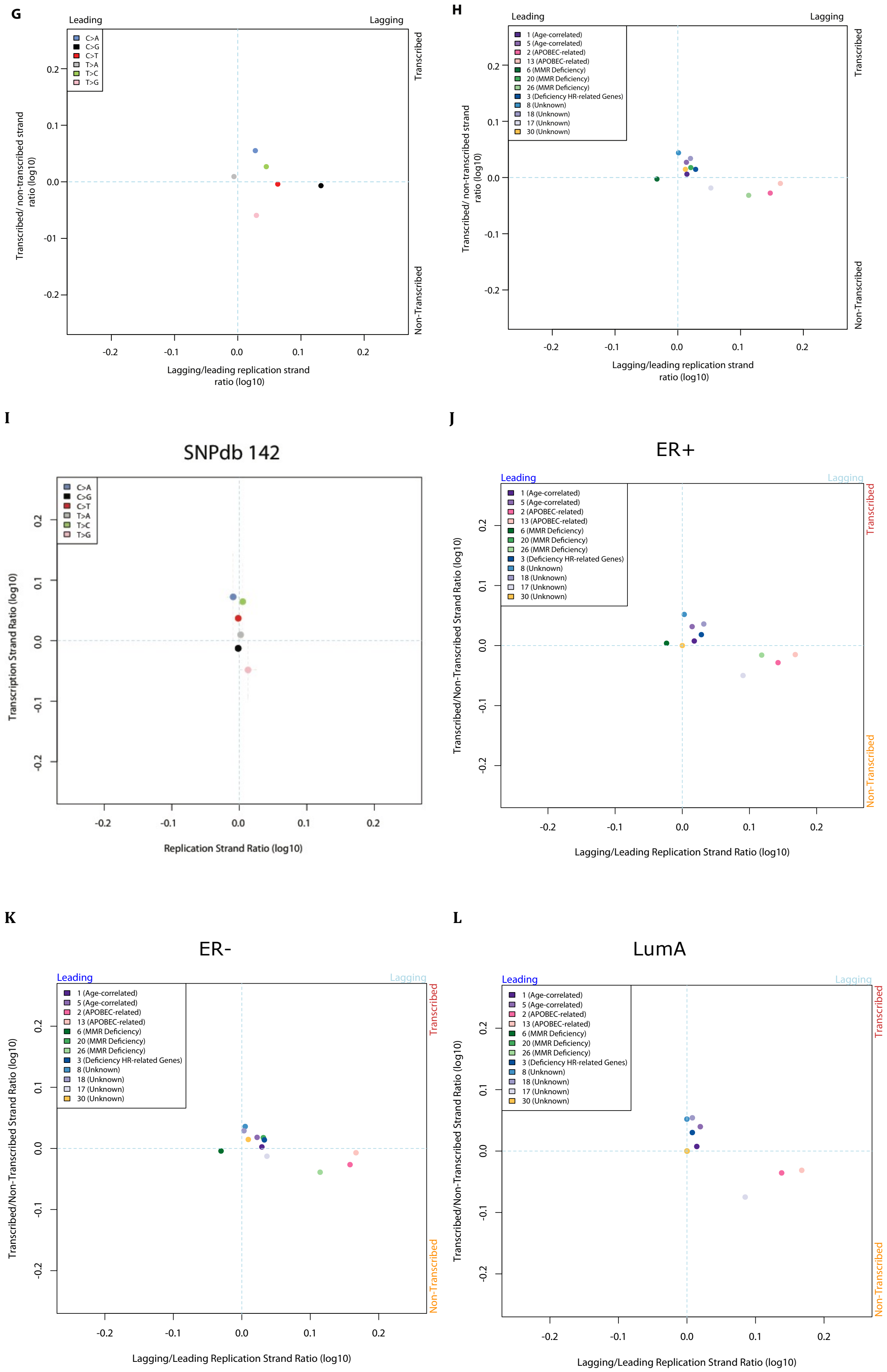
G
Leading
Lagging
C>A
C>G
C>T
T>A
T>C
T>G
Transcribed
Non-Transcribed
Transcribed/ non-transcribed strand ratio (log10)
Lagging/leading replication strand ratio (log10)
H
Leading
Lagging
1 (Age-correlated)
5 (Age-correlated)
2 (APOBEC-related)
13 (APOBEC-related)
6 (MMR Deficiency)
20 (MMR Deficiency)
26 (MMR Deficiency)
3 (Deficiency HR-related Genes)
8 (Unknown)
18 (Unknown)
17 (Unknown)
30 (Unknown)
Transcribed
Non-Transcribed
Transcribed/ non-transcribed strand ratio (log10)
Lagging/leading replication strand ratio (log10)
I
SNPdb 142
C>A
C>G
C>T
T>A
T>C
T>G
Transcription Strand Ratio (log10)
Replication Strand Ratio (log10)
J
ER+
Leading
Lagging
Transcribed
Non-Transcribed
Transcribed/Non-Transcribed Strand Ratio (log10)
Lagging/Leading Replication Strand Ratio (log10)
K
ER-
Leading
Lagging
Transcribed
Non-Transcribed
Transcribed/Non-Transcribed Strand Ratio (log10)
Lagging/Leading Replication Strand Ratio (log10)
L
LumA
Leading
Lagging
Transcribed
Non-Transcribed
Transcribed/Non-Transcribed Strand Ratio (log10)
Lagging/Leading Replication Strand Ratio (log10)

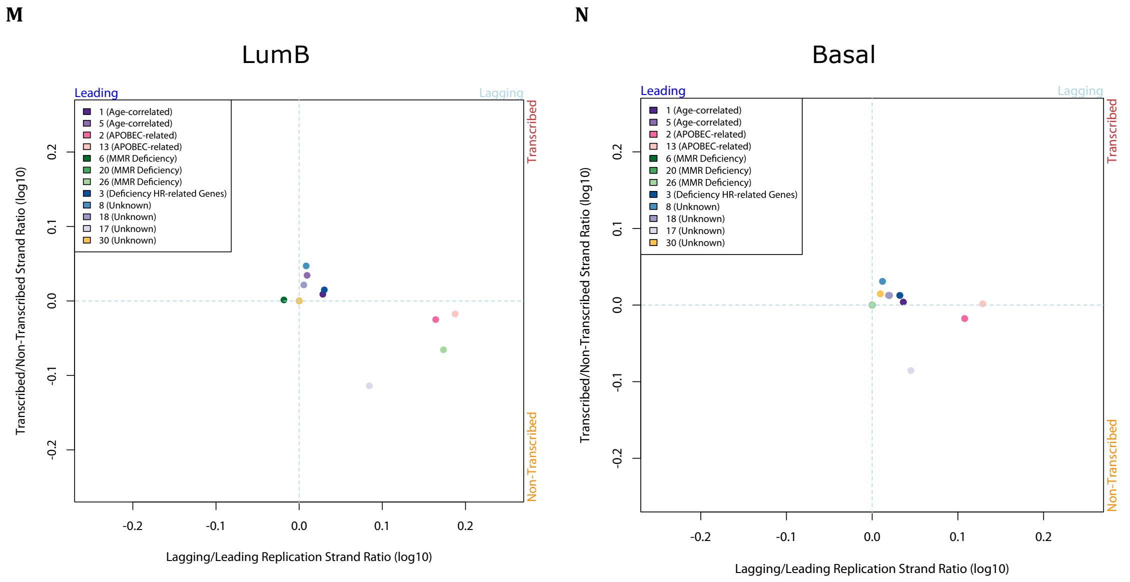

Supplementary Fig. 2 | Characterization of replication strands. A, Overview of the approach used for the identification of the replication strands based on Repli-seq data signal. The figure shows an example of a processed Repli-seq signal, where the local maxima is highlighted with a vertical dashed grey line. This is the point that is replicated before other local regions and acts as a replication initiation point. Peaks in these processed signal data are therefore replicated earlier than troughs. Because of the directionality of DNA synthesis, replicative strands can be inferred. From the local maxima, the direction of the replication fork moving leftwards means that the 3' to 5' DNA strand to the left of the local maxima acts as a template for 5' to 3' DNA synthesis of nascent strands, thus is the template for the leading strand. Conversely, for the replication fork moving rightwards (right of the local maxima), the 5' to 3' DNA strand is the template for the lagging strand. **B,** Smoothened profile of replication time signal obtained from MCF-7 RepliSeq data. The x-axis shows the genomic position of the region in Mb (in this example, chr10:16,437,499-16,437,499), and the y-axis reports the observed replication time signal. The dashed vertical lines highlight distance intervals of 25 Kb. Between the earliest and latest domains, regions with several mini-peaks/valleys are noted and are excluded from reference coordinates. Only long stretches of consistent early-to-late or late-to-early transition zones are used in defining replication strands (highlighted in red). **C-**

D, Size, base content, and distribution of transcribed and non-transcribed strands for the p to q-leading and p to q-lagging replication strands. **E,** Distribution of replication and transcription strand ratios (in log10 scale) for the six pyrimidine mutation classes (each dot represents a sample). **F,** Distribution of replication and transcription strand ratios (in log10 scale) for the twelve signatures (each dot represents a sample). **G,** Distribution of the replication and transcription strand ratios (in log10 scale) of the six pyrimidine mutation classes **H,** of the twelve base substitution signatures **I,** and of variations from dbSNP human build 142. **J-N.** Replication and transcription strand bias for the twelve base substitution signatures, where samples were grouped together by using ER status (ER positive 366 samples and ER negative 194 samples) **J,** and **K,** respectively, and gene expression profiles LumA (101 samples), LumB (75 samples) and Basal (74 samples) **L, M** and **N,** respectively [see [15] for further details on complete cohort].

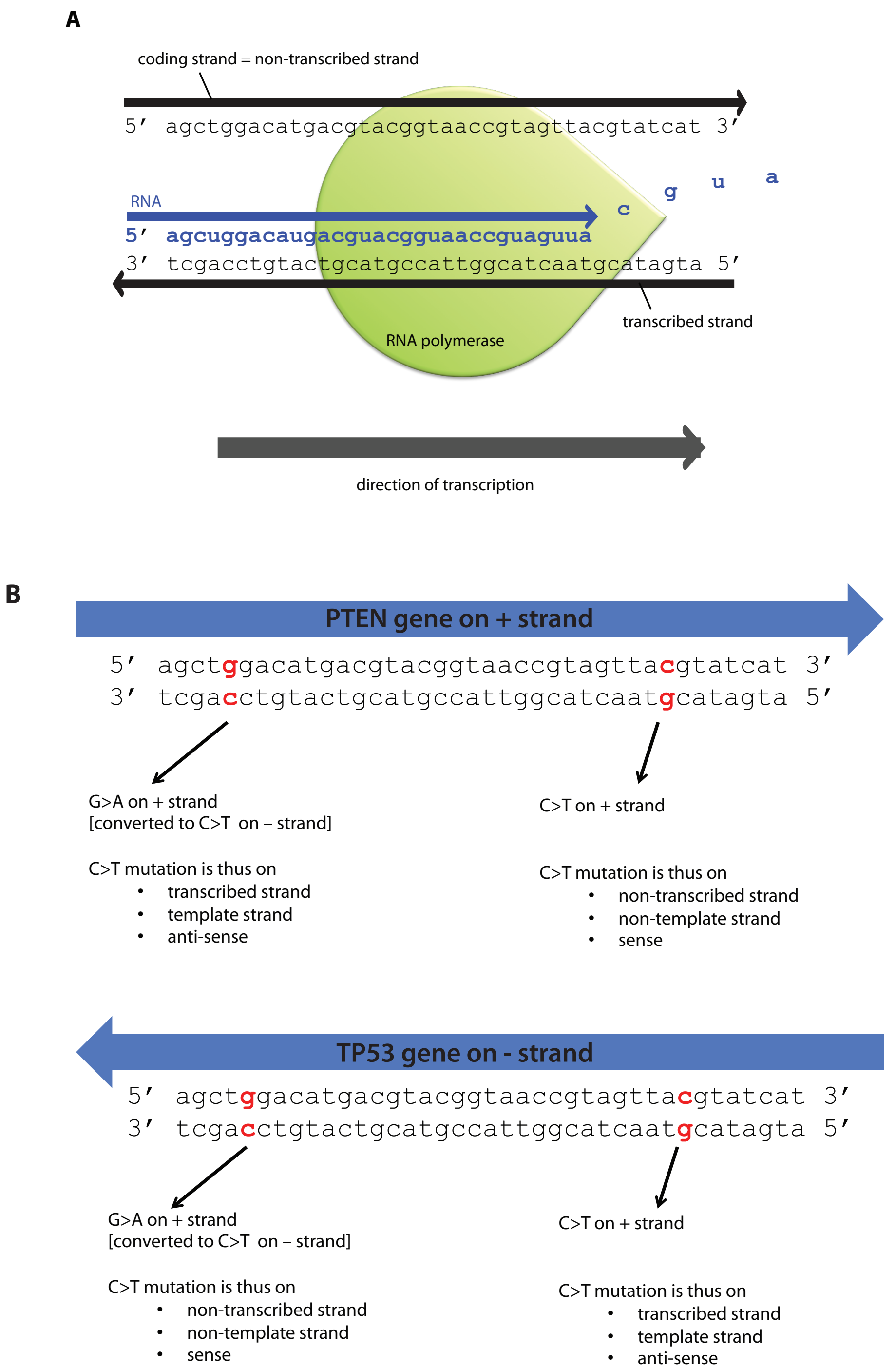

Supplementary Fig. 3 | Characterization of transcriptional strands. A, The nucleotide sequence of transcribed RNA is identical to the sense/non-template/non-transcribed strand, except that U replaces T, and is complementary to that of the anti-sense/template/transcribed strand. **B** Example of mutation presented in pyrimidine context and assigned to transcriptional strands

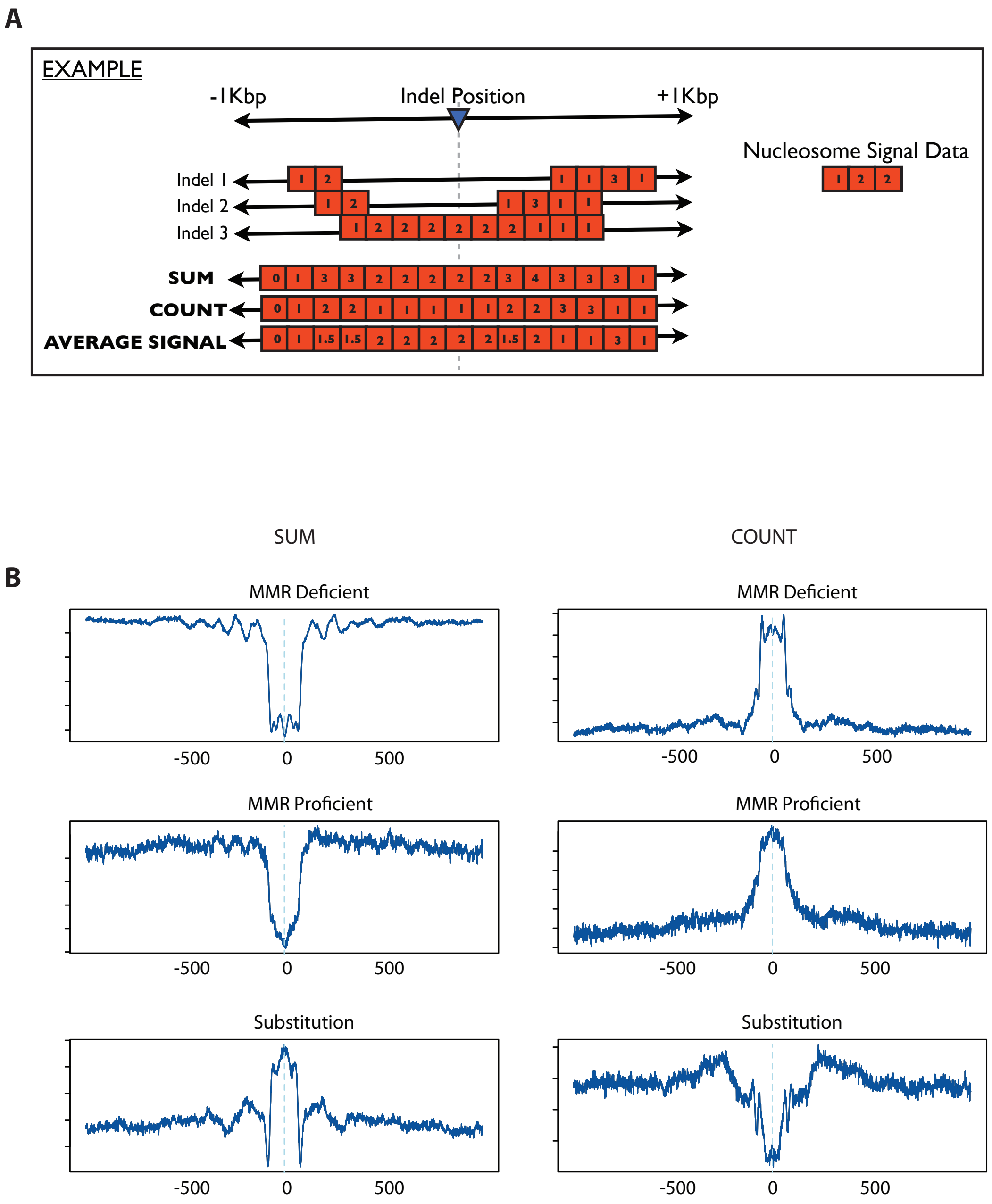

Supplementary Fig. 4 | Relationships between base substitution signatures and nucleosome occupancy. A, Overview of the approach used to compute the relationship between mutations (within individual signatures) and nucleosome occupancy. For all mutations in any given signature, the signal obtained from the MNase experiments (indicating the putative presence of a nucleosome) was summed and divided by the total number of mutations relevant to that signature, for a window of 2kb around each mutation (Supplementary Fig. 4A provides a diagram and example) providing an average signal (y-axis of Figure 3). Thus, the mutations that account for each signature are at the centermost point of the

windows in Figure 3. **B,** Intermediary steps in obtaining the averaged signal. Left panels show the sum of the signal across all the somatic variants for a given signature, the right panels show the total number or count of somatic variants contributing to the total signal.

A

Early <----- Replication time deciles -----> Late

	1	2	3	4	5	6	7	8	9	10
BG02ES	51.7	25.3	17.5	15.9	14.7	15.3	15.6	18.2	21.4	70.1
BJ	56.7	27.5	19.8	17.4	17.2	16.8	17.9	20.3	28.2	52.6
GM06990	58.6	29.9	23.1	19.4	18.6	16.9	19.9	20.3	25.9	81.2
GM12801	60.9	31.1	23.3	19	17.9	16.3	19.2	18.4	24	81.9
GM12812	53.9	26.8	19.8	18.4	18.2	16.3	18.9	18.8	25.2	80.9
GM12813	58.1	30.3	22.9	20.5	19.5	17.3	19	20.5	25.8	83
GM12878	59.4	29.1	23.1	19.7	18.7	16.8	17.9	20.3	27.9	82.5
HeLa-S3	64.1	32.9	23.2	18.5	17.8	16.6	18.9	22.3	28	82.9
HepG2	64	34.7	26.2	22.2	21.1	20.8	19.9	21.4	26.4	77.6
HUVEC	60.1	30.9	19.3	17.9	16.2	16.3	17.8	20.2	27.2	85
IMR90	56.3	25	18.8	17.1	16	16.2	16.7	19.9	27	79.8
K562	64.6	34.2	24.4	21.2	20.2	18.3	19.1	19.4	24	80.1
SK-N-SH	69	34.8	23.6	18.8	17.4	16.4	15.7	17.5	22.8	74.8

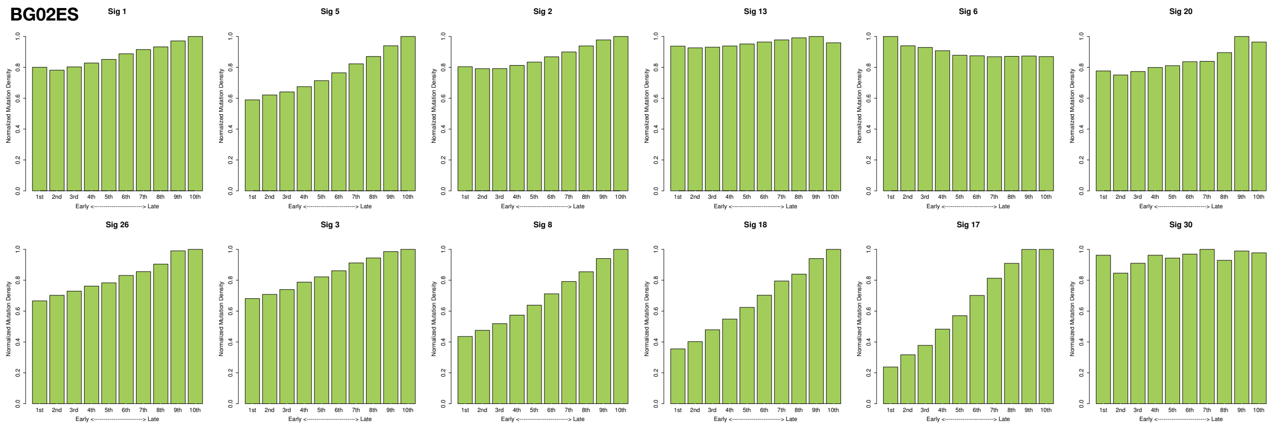

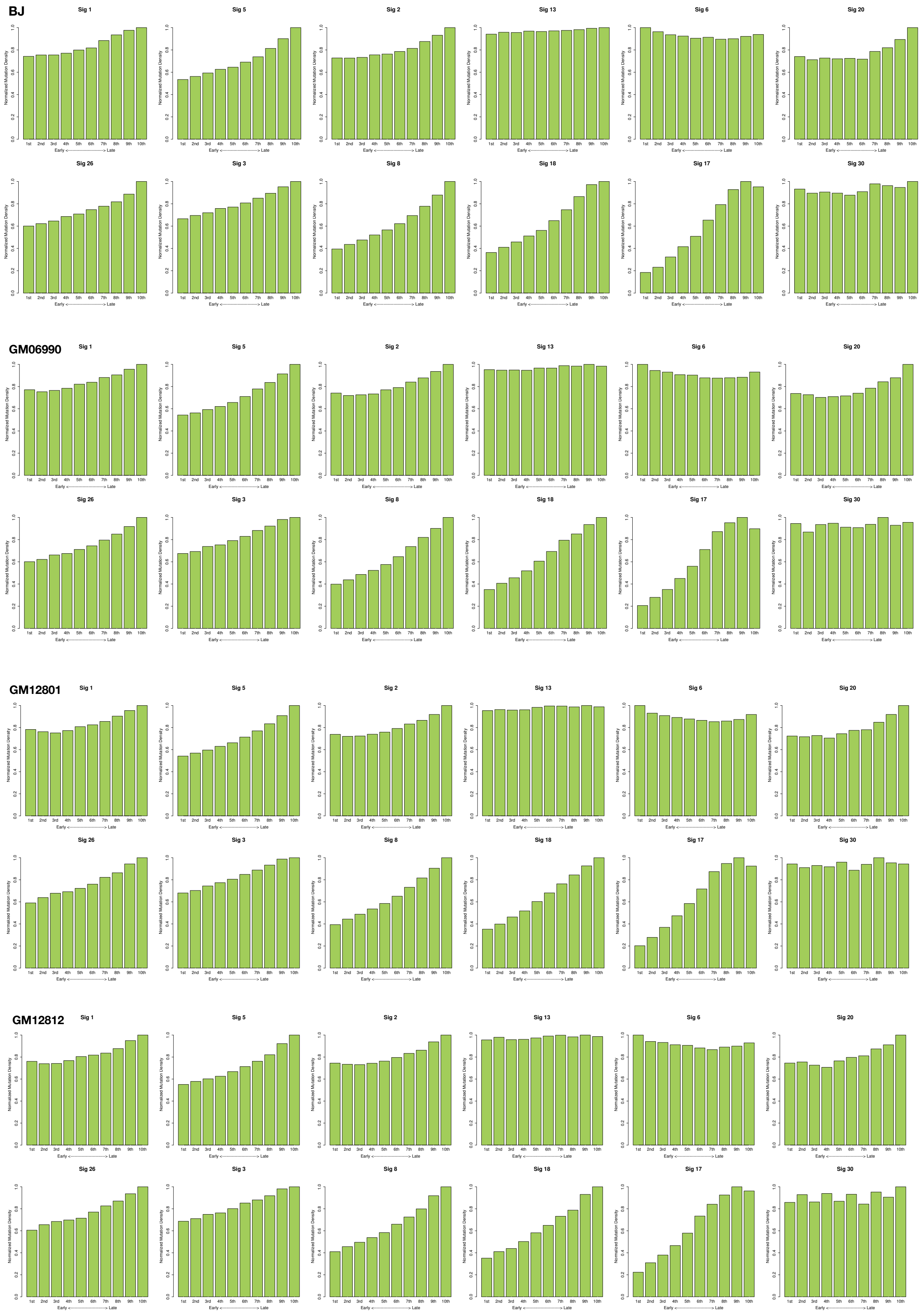
BJ
GM06990
GM12801
GM12812
Sig 1
Sig 5
Sig 2
Sig 13
Sig 6
Sig 20
Sig 26
Sig 3
Sig 8
Sig 18
Sig 17
Sig 30
Normalized Mutation Density
1st
2nd
3rd
4th
5th
6th
7th
8th
9th
10th
Early <----------------------------> Late

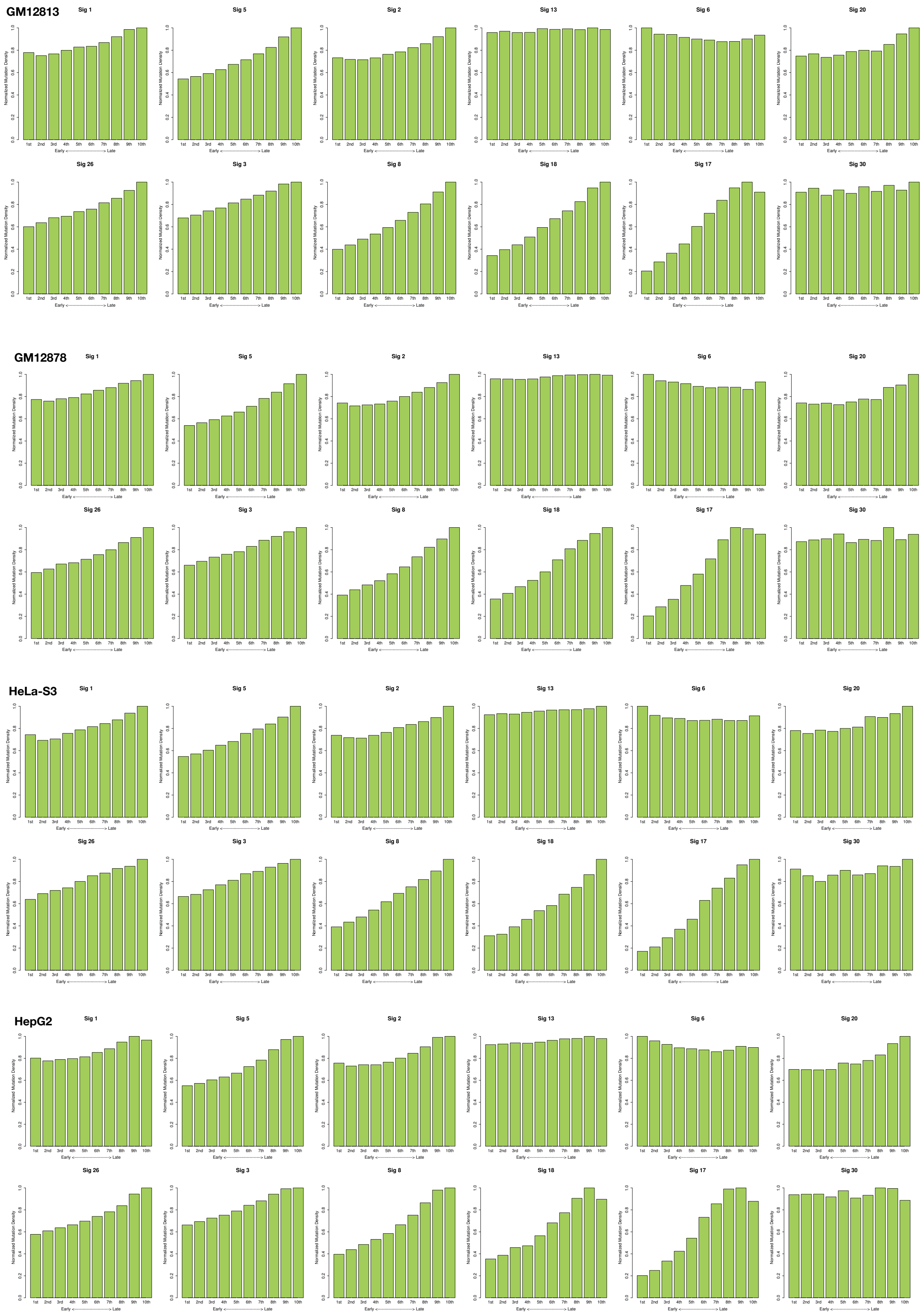
GM12813
Sig 1
Sig 5
Sig 2
Sig 13
Sig 6
Sig 20
Sig 26
Sig 3
Sig 8
Sig 18
Sig 17
Sig 30
GM12878
HeLa-S3
HepG2
Normalized Mutation Density
1st
2nd
3rd
4th
5th
6th
7th
8th
9th
10th
Early
Late

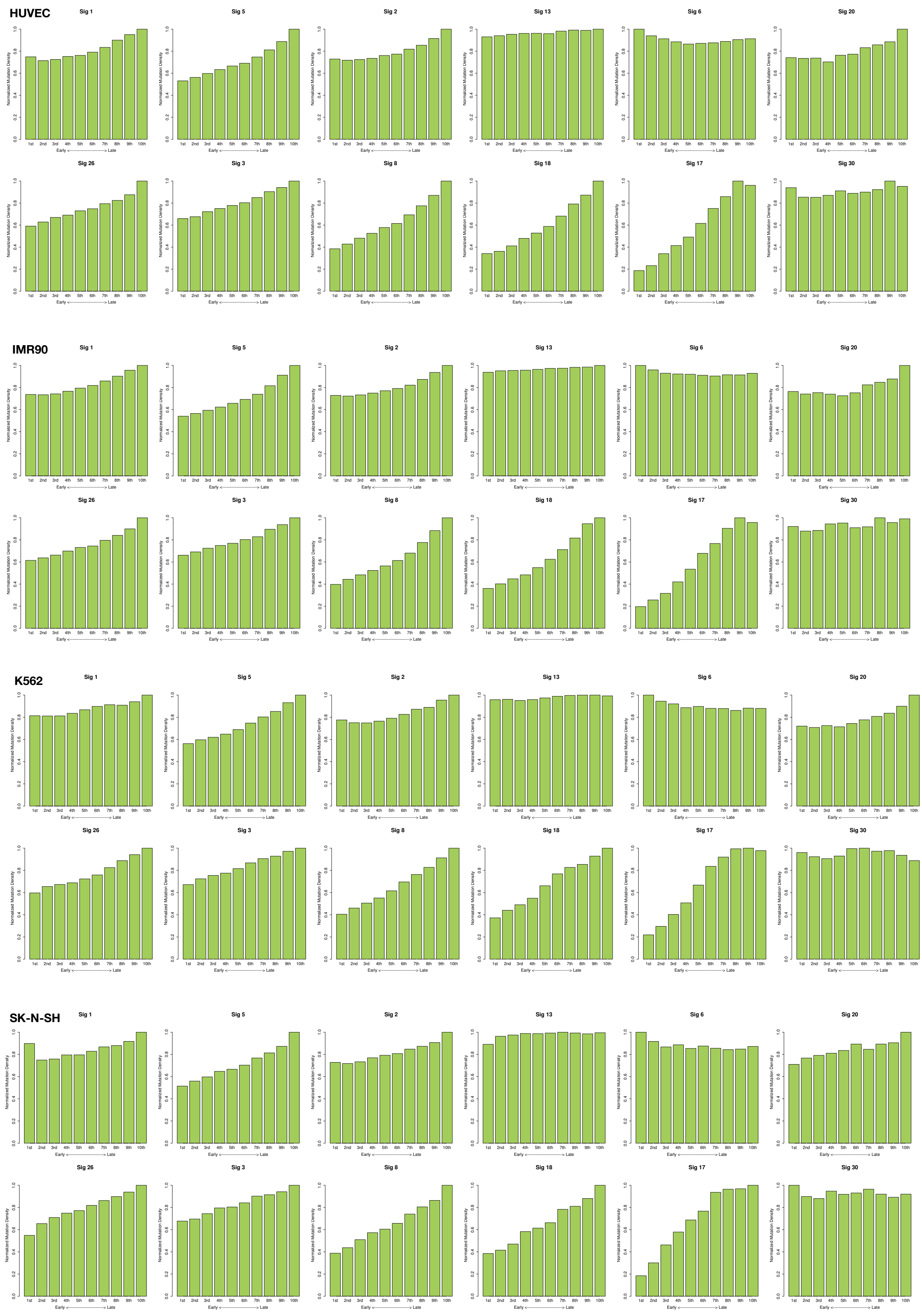
HUVEC
IMR90
K562
SK-N-SH
Sig 1
Sig 5
Sig 2
Sig 13
Sig 6
Sig 20
Sig 26
Sig 3
Sig 8
Sig 18
Sig 17
Sig 30
Normalized Mutation Density
1st 2nd 3rd 4th 5th 6th 7th 8th 9th 10th
Early Late

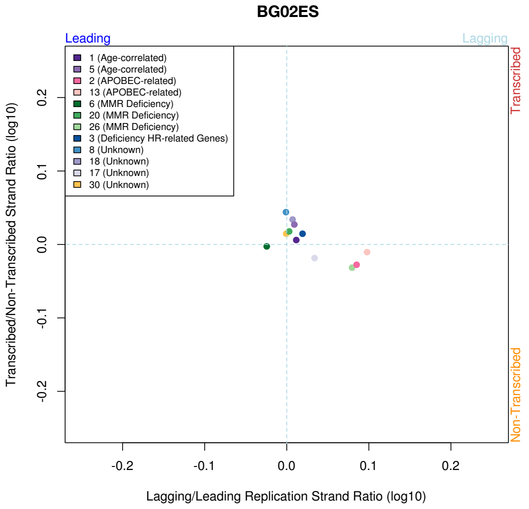
BG02ES
Leading
Lagging
Transcribed
Non-Transcribed
1 (Age-correlated)
5 (Age-correlated)
2 (APOBEC-related)
13 (APOBEC-related)
6 (MMR Deficiency)
20 (MMR Deficiency)
26 (MMR Deficiency)
3 (Deficiency HR-related Genes)
8 (Unknown)
18 (Unknown)
17 (Unknown)
30 (Unknown)
Transcribed/Non-Transcribed Strand Ratio (log10)
Lagging/Leading Replication Strand Ratio (log10)

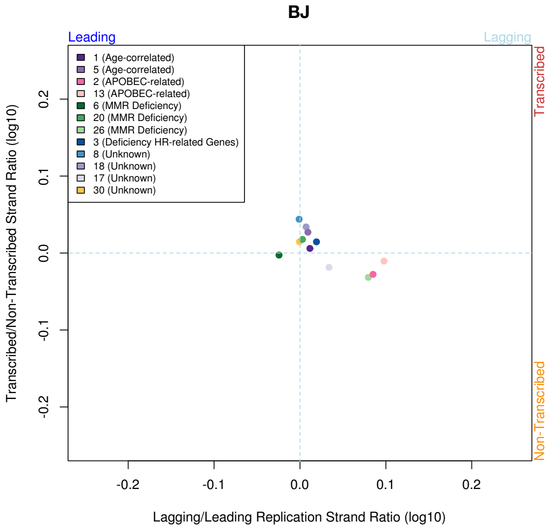
BJ
Leading
Lagging
Transcribed
Non-Transcribed
1 (Age-correlated)
5 (Age-correlated)
2 (APOBEC-related)
13 (APOBEC-related)
6 (MMR Deficiency)
20 (MMR Deficiency)
26 (MMR Deficiency)
3 (Deficiency HR-related Genes)
8 (Unknown)
18 (Unknown)
17 (Unknown)
30 (Unknown)
Transcribed/Non-Transcribed Strand Ratio (log10)
Lagging/Leading Replication Strand Ratio (log10)

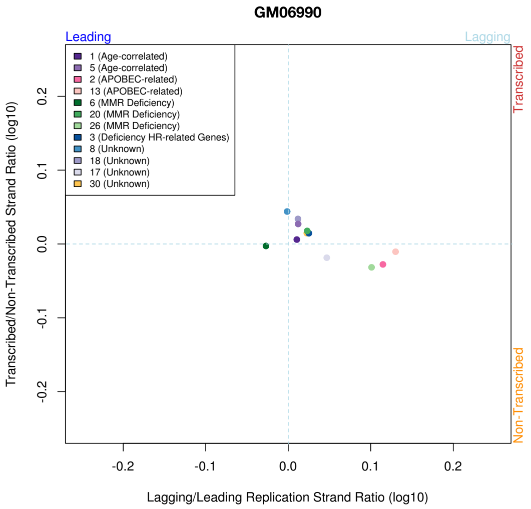
GM06990
Leading
Lagging
Transcribed
Non-Transcribed
1 (Age-correlated)
5 (Age-correlated)
2 (APOBEC-related)
13 (APOBEC-related)
6 (MMR Deficiency)
20 (MMR Deficiency)
26 (MMR Deficiency)
3 (Deficiency HR-related Genes)
8 (Unknown)
18 (Unknown)
17 (Unknown)
30 (Unknown)
Transcribed/Non-Transcribed Strand Ratio (log10)
Lagging/Leading Replication Strand Ratio (log10)

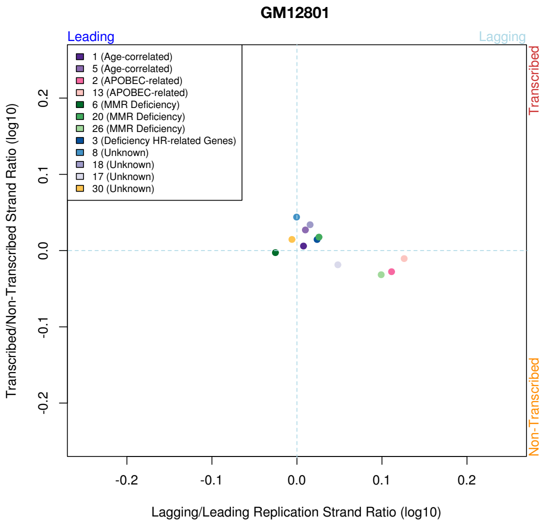
GM12801
Leading
Lagging
Transcribed
Non-Transcribed
1 (Age-correlated)
5 (Age-correlated)
2 (APOBEC-related)
13 (APOBEC-related)
6 (MMR Deficiency)
20 (MMR Deficiency)
26 (MMR Deficiency)
3 (Deficiency HR-related Genes)
8 (Unknown)
18 (Unknown)
17 (Unknown)
30 (Unknown)
Transcribed/Non-Transcribed Strand Ratio (log10)
Lagging/Leading Replication Strand Ratio (log10)

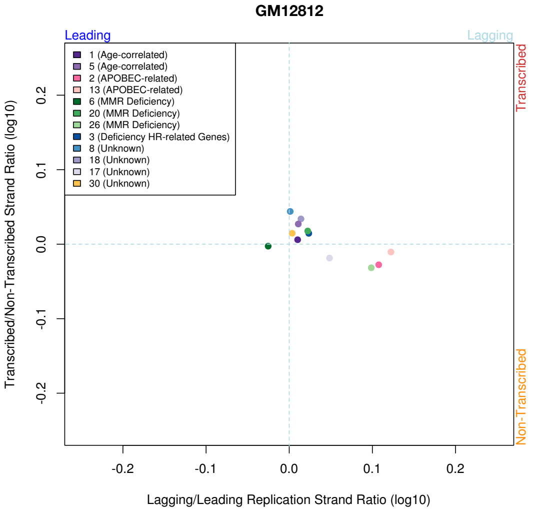
GM12812
Leading
Lagging
Transcribed
Non-Transcribed
1 (Age-correlated)
5 (Age-correlated)
2 (APOBEC-related)
13 (APOBEC-related)
6 (MMR Deficiency)
20 (MMR Deficiency)
26 (MMR Deficiency)
3 (Deficiency HR-related Genes)
8 (Unknown)
18 (Unknown)
17 (Unknown)
30 (Unknown)
Transcribed/Non-Transcribed Strand Ratio (log10)
Lagging/Leading Replication Strand Ratio (log10)

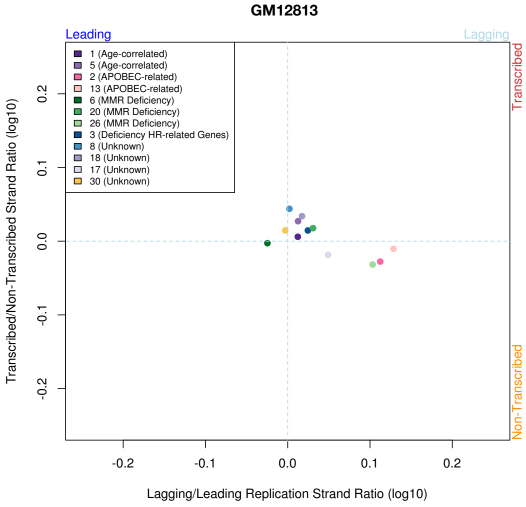
GM12813
Leading
Lagging
Transcribed
Non-Transcribed
1 (Age-correlated)
5 (Age-correlated)
2 (APOBEC-related)
13 (APOBEC-related)
6 (MMR Deficiency)
20 (MMR Deficiency)
26 (MMR Deficiency)
3 (Deficiency HR-related Genes)
8 (Unknown)
18 (Unknown)
17 (Unknown)
30 (Unknown)
Transcribed/Non-Transcribed Strand Ratio (log10)
Lagging/Leading Replication Strand Ratio (log10)

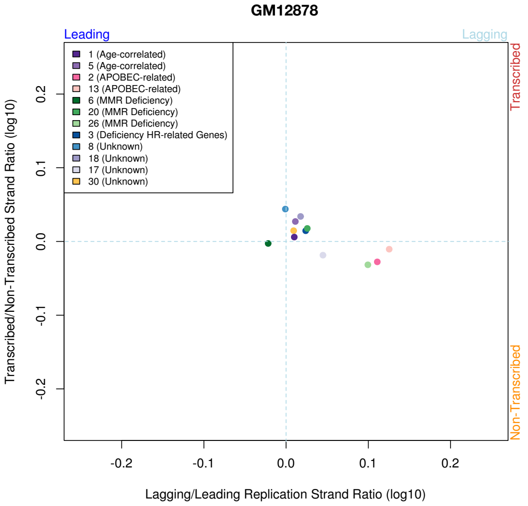
GM12878
Leading
Lagging
1 (Age-correlated)
5 (Age-correlated)
2 (APOBEC-related)
13 (APOBEC-related)
6 (MMR Deficiency)
20 (MMR Deficiency)
26 (MMR Deficiency)
3 (Deficiency HR-related Genes)
8 (Unknown)
18 (Unknown)
17 (Unknown)
30 (Unknown)
Transcribed
Non-Transcribed
Transcribed/Non-Transcribed Strand Ratio (log10)
Lagging/Leading Replication Strand Ratio (log10)
-0.2
-0.1
0.0
0.1
0.2

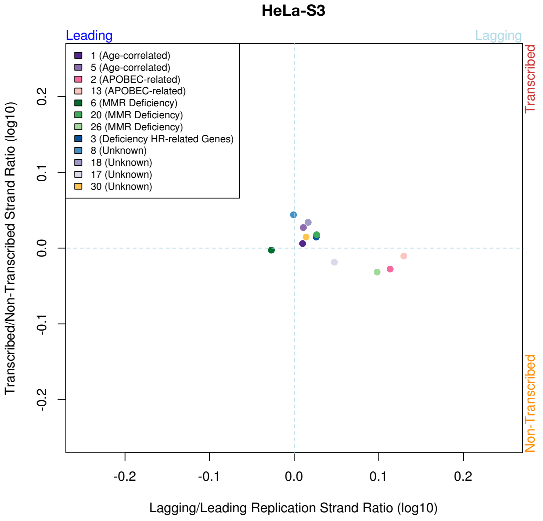
HeLa-S3
Leading
Lagging
1 (Age-correlated)
5 (Age-correlated)
2 (APOBEC-related)
13 (APOBEC-related)
6 (MMR Deficiency)
20 (MMR Deficiency)
26 (MMR Deficiency)
3 (Deficiency HR-related Genes)
8 (Unknown)
18 (Unknown)
17 (Unknown)
30 (Unknown)
Transcribed
Non-Transcribed
Transcribed/Non-Transcribed Strand Ratio (log10)
Lagging/Leading Replication Strand Ratio (log10)
-0.2
-0.1
0.0
0.1
0.2

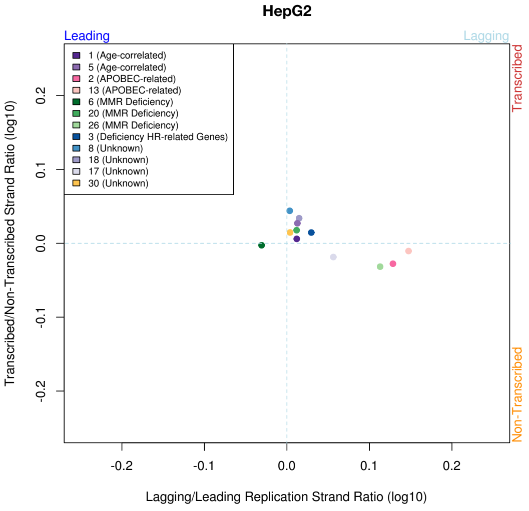
HepG2
Leading
Lagging
1 (Age-correlated)
5 (Age-correlated)
2 (APOBEC-related)
13 (APOBEC-related)
6 (MMR Deficiency)
20 (MMR Deficiency)
26 (MMR Deficiency)
3 (Deficiency HR-related Genes)
8 (Unknown)
18 (Unknown)
17 (Unknown)
30 (Unknown)
Transcribed
Non-Transcribed
Transcribed/Non-Transcribed Strand Ratio (log10)
Lagging/Leading Replication Strand Ratio (log10)
-0.2
-0.1
0.0
0.1
0.2

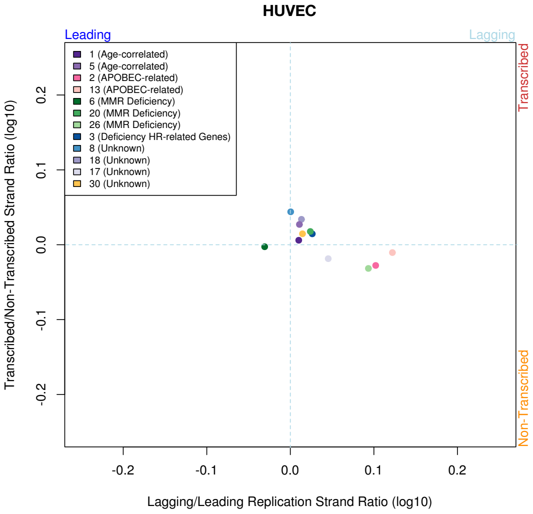
HUVEC
Leading
Lagging
1 (Age-correlated)
5 (Age-correlated)
2 (APOBEC-related)
13 (APOBEC-related)
6 (MMR Deficiency)
20 (MMR Deficiency)
26 (MMR Deficiency)
3 (Deficiency HR-related Genes)
8 (Unknown)
18 (Unknown)
17 (Unknown)
30 (Unknown)
Transcribed
Non-Transcribed
Transcribed/Non-Transcribed Strand Ratio (log10)
Lagging/Leading Replication Strand Ratio (log10)
-0.2
-0.1
0.0
0.1
0.2

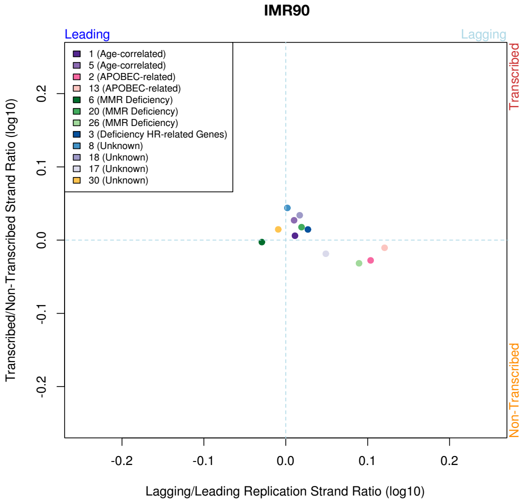
IMR90
Leading
Lagging
1 (Age-correlated)
5 (Age-correlated)
2 (APOBEC-related)
13 (APOBEC-related)
6 (MMR Deficiency)
20 (MMR Deficiency)
26 (MMR Deficiency)
3 (Deficiency HR-related Genes)
8 (Unknown)
18 (Unknown)
17 (Unknown)
30 (Unknown)
Transcribed
Non-Transcribed
Transcribed/Non-Transcribed Strand Ratio (log10)
Lagging/Leading Replication Strand Ratio (log10)
-0.2
-0.1
0.0
0.1
0.2

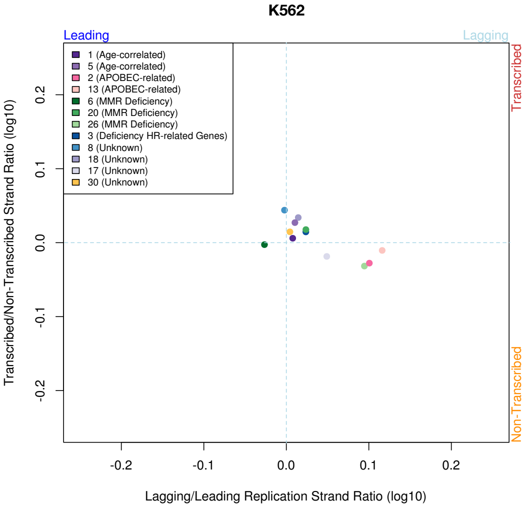
K562
Leading
Lagging
1 (Age-correlated)
5 (Age-correlated)
2 (APOBEC-related)
13 (APOBEC-related)
6 (MMR Deficiency)
20 (MMR Deficiency)
26 (MMR Deficiency)
3 (Deficiency HR-related Genes)
8 (Unknown)
18 (Unknown)
17 (Unknown)
30 (Unknown)
Transcribed
Non-Transcribed
Transcribed/Non-Transcribed Strand Ratio (log10)
Lagging/Leading Replication Strand Ratio (log10)
-0.2
-0.1
0.0
0.1
0.2

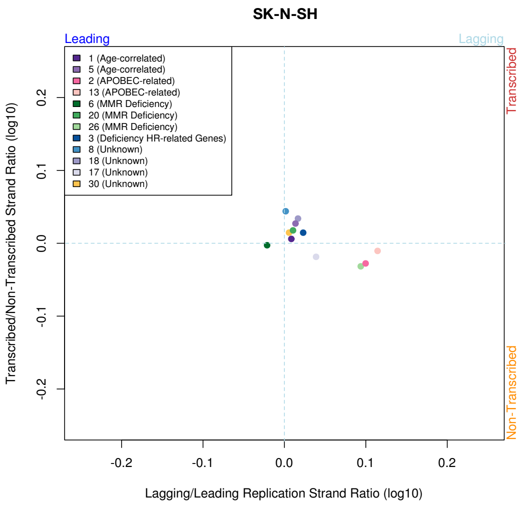

Supplementary Fig. 5 | Comparison between MCF7 and publicly available datasets on ENCODE. A, Details of the replication time domain shared between MCF-7 and other cell lines. The figure reports the percentage of genome that MCF-7 shares with the other thirteen cell lines (rows) in each computed decile (columns). Deciles are numbered from 1 (earliest decile) to 10 (latest decile). A large proportion of the earliest (average 59.8%) and latest (average 77.9%) replication domains are shared between all thirteen cell lines and MCF7. Subsequent pages demonstrate results of analysis of replication time and replication and transcriptional strand biases for the remaining thirteen cell lines. Note the striking similarity between cell lines, particularly for the signatures with the strongest biological signals.

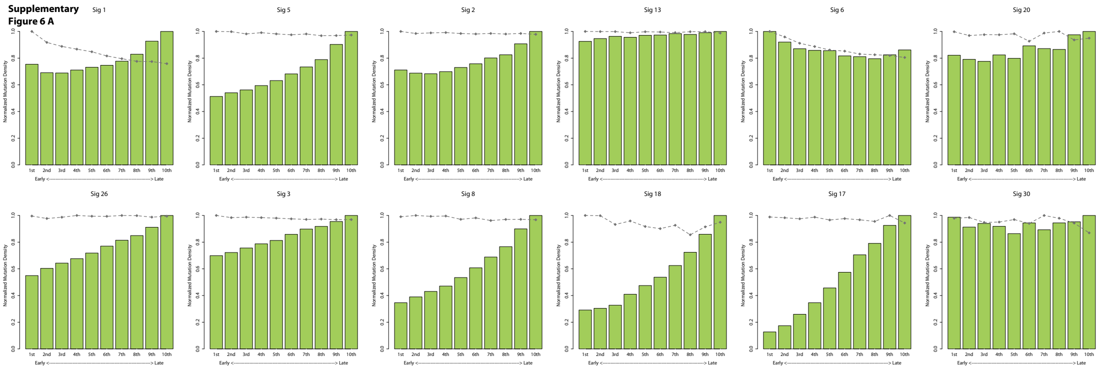

Supplementary Figure 6

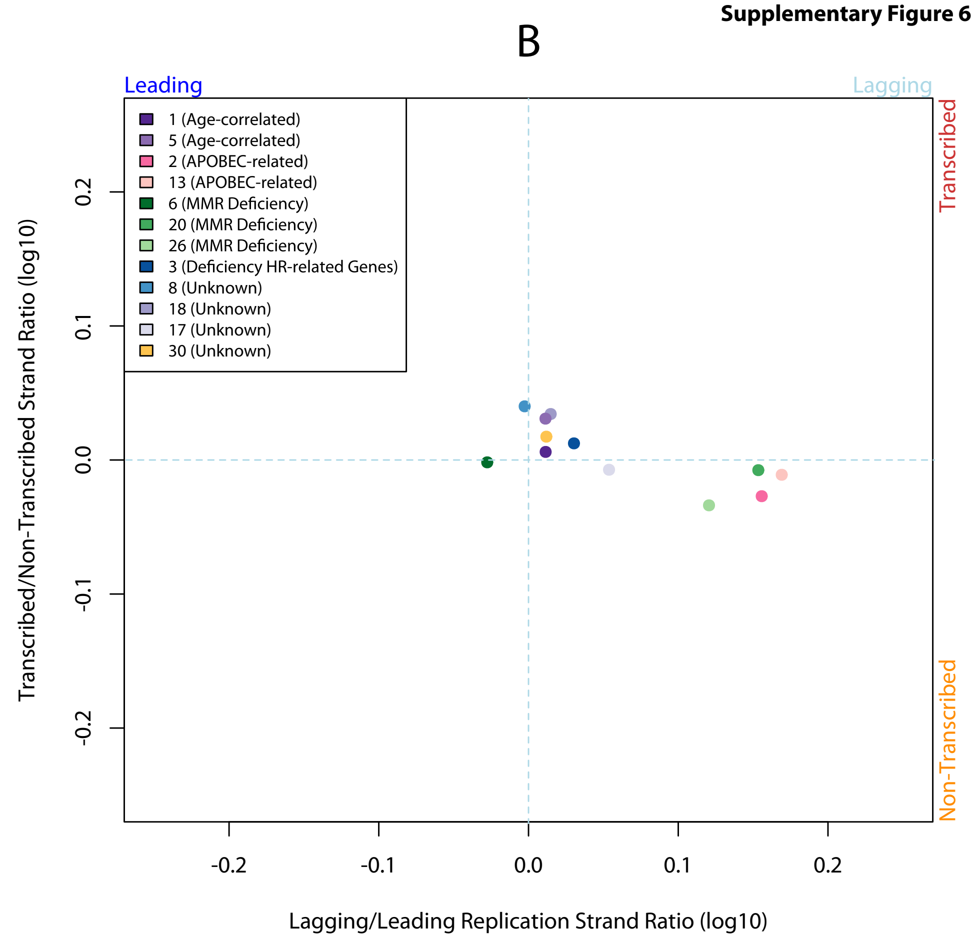

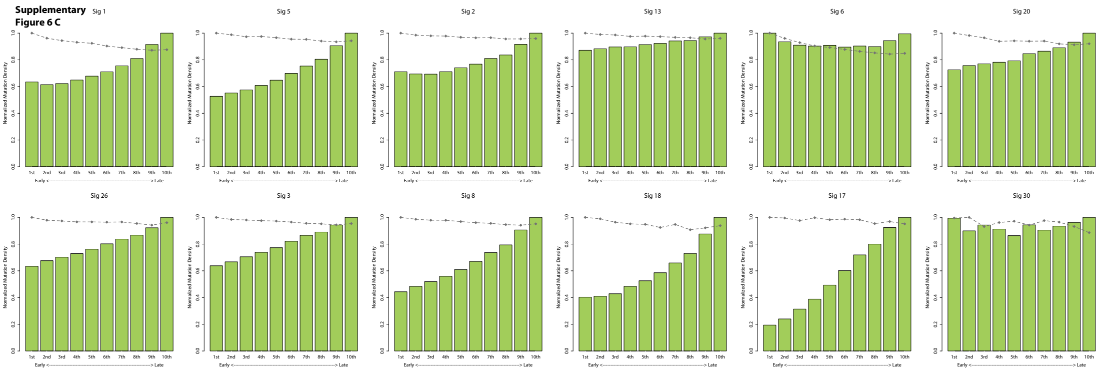

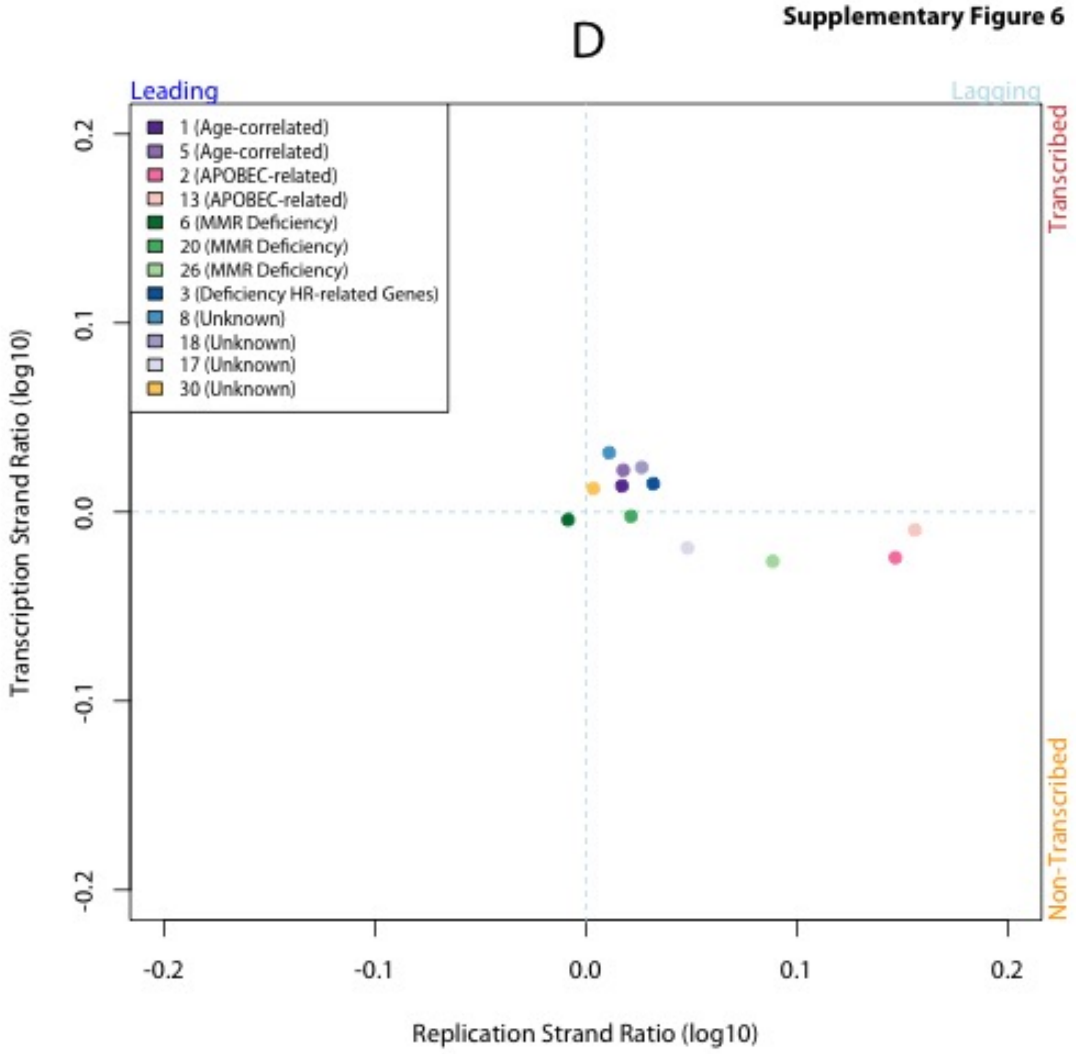

Supplementary Fig. 6 | Replication time, and replication vs transcription strand analyses performed by using 'maximum likelihood with probability threshold' and 'belief propagation' approaches. A-B, Replication time and replication vs transcription strand analyses performed by using maximum likelihood with probability threshold (signatures with a posteriori probability lower or equal to 0.5 were filtered out). **C-D,** Replication time and replication vs transcription strand analyses performed by using belief propagation. Comparison of these plots with the ones reported in the main manuscript (Figure 1 and Figure 2) shows that the main results were consistent across the different methodologies.

	Replication Strand									
	Observed			Binomial Test		Simulated			Binomial Test	
	Lagging	Leading	Ratio (log10)	p-value	Confidence Interval	Lagging	Leading	Ratio (log10)	p-value	Confidence Interval
C>A	234,807	220,260	0.0278	3.9E-103	0.5145 - 0.5174	226,077	226,444	-0.0007	4.4E-01	0.4972 - 0.5012
C>G	348,626	257,477	0.1316	2.5E-323	0.5739 - 0.5764	297,650	297,785	-0.0002	3.8E-01	0.4975 - 0.5010
C>T	553,456	478,395	0.0633	3.5E-323	0.5354 - 0.5373	509,530	510,047	-0.0004	8.3E-01	0.4988 - 0.5015
T>A	144,875	146,900	-0.0060	1.8E-04	0.4947 - 0.4983	145,077	145,280	-0.0006	4.4E-01	0.4985 - 0.5035
T>C	222,452	200,497	0.0451	6.4E-250	0.5244 - 0.5275	210,120	210,067	0.0001	3.4E-01	0.4989 - 0.5031
T>G	96,652	90,312	0.0295	1.1E-48	0.5147 - 0.5192	92,423	93,371	-0.0044	2.9E-01	0.4952 - 0.5014
1	133,244	128,873	0.0145	1.4E-17	0.5064 - 0.5103	130,689	130,792	-0.0003	3.6E-01	0.4986 - 0.5038
5	292,405	283,323	0.0137	5.2E-33	0.5066 - 0.5092	286,766	288,687	-0.0029	4.4E-01	0.4975 - 0.5011
2	231,923	165,221	0.1473	1.9E-323	0.5824 - 0.5855	195,348	195,663	-0.0007	3.2E-01	0.4989 - 0.5032
13	295,962	203,155	0.1634	2.5E-323	0.5916 - 0.5943	245,661	244,090	0.0028	1.4E-01	0.4966 - 0.5005
6	61,025	65,877	-0.0332	3.1E-42	0.4781 - 0.4836	62,606	62,259	0.0024	8.7E-01	0.4959 - 0.5034
20	12,501	11,921	0.0206	2.1E-04	0.5056 - 0.5182	11,879	12,035	-0.0057	2.2E-01	0.4968 - 0.5143
26	64,882	50,027	0.1129	9.9E-324	0.5618 - 0.5675	56,664	56,933	-0.0021	8.5E-01	0.4964 - 0.5044
3	326,784	306,105	0.0284	5.7E-149	0.5151 - 0.5176	311,298	312,595	-0.0018	7.8E-01	0.4985 - 0.5019
8	147,483	147,105	0.0011	4.9E-01	0.4988 - 0.5024	146,506	146,580	-0.0002	7.0E-01	0.4970 - 0.5020
18	17,357	16,569	0.0202	1.9E-05	0.5063 - 0.5169	16,916	17,100	-0.0047	6.1E-01	0.4946 - 0.5092
17	13,396	11,866	0.0527	6.4E-22	0.5241 - 0.5365	12,710	12,461	0.0086	3.7E-01	0.4877 - 0.5046
30	3,906	3,799	0.0121	2.3E-01	0.4957 - 0.5182	3,834	3,799	0.0040	5.3E-01	0.4795 - 0.5104

	Transcription Strand									
	Observed			Binomial Test		Simulated			Binomial Test	
	Transcribed	Non-Transcribed	Ratio (log10)	p-value	Confidence Interval	Transcribed	Non-Transcribed	Ratio (log10)	p-value	Confidence Interval
C>A	108,109	95,222	0.0551	5.9E-179	0.4661 - 0.4705	121,451	121,067	0.0014	4.4E-01	0.4972 - 0.5012
C>G	154,073	156,507	-0.0068	3.8E-05	0.5022 - 0.5057	159,381	158,884	0.0014	3.8E-01	0.4975 - 0.5010
C>T	253,176	255,535	-0.0040	9.5E-04	0.5009 - 0.5037	274,835	274,999	-0.0003	8.3E-01	0.4988 - 0.5015
T>A	65,055	63,679	0.0093	2.5E-04	0.4919 - 0.4974	76,841	77,143	-0.0017	4.4E-01	0.4985 - 0.5035
T>C	102,486	96,356	0.0268	2.2E-42	0.4824 - 0.4868	111,166	111,614	-0.0017	3.4E-01	0.4989 - 0.5031
T>G	41,005	47,013	-0.0594	1.6E-90	0.5308 - 0.5374	49,622	49,288	0.0029	2.9E-01	0.4952 - 0.5014
1	63,651	62,781	0.0060	7.3E-02	0.4938 - 0.4993	71,683	72,029	-0.0021	3.6E-01	0.4986 - 0.5038
5	140,740	132,227	0.0271	1.2E-58	0.4825 - 0.4863	153,736	153,306	0.0012	4.4E-01	0.4975 - 0.5011
2	96,301	102,638	-0.0277	8.3E-45	0.5137 - 0.5181	103,929	104,387	-0.0019	3.2E-01	0.4989 - 0.5032
13	126,214	129,298	-0.0105	7.5E-09	0.5041 - 0.5080	130,901	130,152	0.0025	1.4E-01	0.4966 - 0.5005
6	33,586	33,794	-0.0027	6.0E-01	0.4978 - 0.5053	34,093	34,049	0.0006	8.7E-01	0.4959 - 0.5034
20	6,364	6,110	0.0177	9.4E-02	0.4810 - 0.4986	6,272	6,412	-0.0096	2.2E-01	0.4968 - 0.5143
26	27,794	29,895	-0.0316	1.8E-17	0.5141 - 0.5223	30,217	30,264	-0.0007	8.5E-01	0.4964 - 0.5044
3	148,331	143,445	0.0145	1.4E-18	0.4898 - 0.4934	166,407	166,569	-0.0004	7.8E-01	0.4985 - 0.5019
8	66,380	59,990	0.0440	3.6E-71	0.4720 - 0.4775	78,124	77,969	0.0009	7.0E-01	0.4970 - 0.5020
18	7,505	6,941	0.0339	1.7E-05	0.4723 - 0.4887	9,100	9,169	-0.0033	6.1E-01	0.4946 - 0.5092
17	5,047	5,268	-0.0186	9.4E-02	0.5010 - 0.5204	6,779	6,675	0.0067	3.7E-01	0.4877 - 0.5046
30	1,991	1,925	0.0146	6.0E-01	0.4758 - 0.5074	2,055	2,014	0.0088	5.3E-01	0.4795 - 0.5104

Supplementary Table 1 | Comparison between observed and simulated replication and transcriptional strand bias. For each mutation and signature, we computed the replication and transcription strand ratio (see Methods). The ratios were computed for both observed and simulated data (see Methods for more details on the approach used to generate the simulate data). Unlike observed data, simulated data ratios (log10) were always close to 0, that is, simulated data tended not to show any replication or transcription strand bias. Thus the null hypothesis expects a 50:50 distribution with respect to transcriptional and replication strands. A binomial test with the expected probability of success of 0.5 was used to compare the number of mutations observed on the different strands, obtaining both *p*-values and 95% confidence intervals.

Processive Group Length	Signature 1 Number of Groups	Signature 1 Mut Density (per Mb)	Signature 5 Number of Groups	Signature 5 Mut Density (per Mb)	Signature 2 Number of Groups	Signature 2 Mut Density (per Mb)	Signature 13 Number of Groups	Signature 13 Mut Density (per Mb)	Signature 6 Number of Groups	Signature 6 Mut Density (per Mb)	Signature 20 Number of Groups	Signature 20 Mut Density (per Mb)
2	21,792	4.1	62,708	4.2	48,104	33.8	59,777	31.8	16,233	70.3	2,201	104.1
3	2,774	2.2	9,463	2.3	12,015	26.7	15,535	22.6	3,752	43.4	279	60.8
4	433	1.7	1,615	1.7	3,485	25.4	5,035	20.9	1,067	39.8	27	44.2
5	76	1.3	320	1.4	1,079	23.3	1,831	19.8	320	33.2	3	71.6
6	16	1.9	65	1.3	393	24.5	753	17.9	91	30.8	1	17.3
7	5	1.7	17	7.0	134	22.7	346	19.6	27	30.8	0	0.0
8	1	0.6	2	18.2	65	28.3	159	18.3	14	31.9	0	0.0
9	0	0.0	2	0.5	27	33.2	86	16.4	5	23.4	0	0.0
10	0	0.0	1	0.4	11	79.6	48	17.6	3	46.2	0	0.0
11	0	0.0	0	0.0	8	25.3	24	15.3	1	27.1	0	0.0
12	0	0.0	0	0.0	4	31.4	18	16.2	0	0.0	0	0.0
13	0	0.0	0	0.0	1	397.0	10	17.3	0	0.0	0	0.0
14	0	0.0	0	0.0	0	0.0	5	18.6	0	0.0	0	0.0
15	0	0.0	0	0.0	0	0.0	5	12.2	0	0.0	0	0.0
16	0	0.0	0	0.0	2	371.7	6	19.2	0	0.0	0	0.0
17	0	0.0	0	0.0	0	0.0	2	17.7	0	0.0	0	0.0
18	0	0.0	0	0.0	0	0.0	0	0.0	0	0.0	0	0.0
19	0	0.0	0	0.0	0	0.0	2	17.6	0	0.0	0	0.0

Processive Group Length	Signature 26 Number of Groups	Signature 26 Mut Density (per Mb)	Signature 3 Number of Groups	Signature 3 Mut Density (per Mb)	Signature 8 Number of Groups	Signature 8 Mut Density (per Mb)	Signature 18 Number of Groups	Signature 18 Mut Density (per Mb)	Signature 17 Number of Groups	Signature 17 Mut Density (per Mb)	Signature 30 Number of Groups	Signature 30 Mut Density (per Mb)
2	14,468	43.6	80,249	12.8	23,914	11.4	2,412	6.6	2,924	34.9	1,064	16.6
3	3,328	21.2	15,010	7.7	2,740	7.4	263	4.1	861	25.9	272	10.2
4	950	12.9	2,996	6.6	334	6.4	39	3.6	314	23.7	70	8.5
5	292	9.9	646	5.9	51	8.2	4	6.2	119	24.2	16	7.9
6	96	9.6	141	5.2	8	9.7	0	0.0	55	22.3	6	10.6
7	36	8.7	31	5.6	4	9.3	0	0.0	22	22.7	2	5.9
8	12	4.3	3	2.6	0	0.0	0	0.0	13	17.8	0	0.0
9	3	6.9	2	9.0	0	0.0	1	9.3	5	29.8	0	0.0
10	0	0.0	1	2.4	0	0.0	0	0.0	1	43.0	0	0.0
11	2	33.4	0	0.0	0	0.0	0	0.0	0	0.0	0	0.0
12	0	0.0	0	0.0	0	0.0	0	0.0	0	0.0	0	0.0
13	0	0.0	0	0.0	0	0.0	0	0.0	0	0.0	0	0.0
14	1	7.2	0	0.0	0	0.0	0	0.0	0	0.0	0	0.0
15	0	0.0	0	0.0	0	0.0	0	0.0	0	0.0	0	0.0
16	0	0.0	0	0.0	0	0.0	0	0.0	0	0.0	0	0.0
17	0	0.0	0	0.0	0	0.0	0	0.0	0	0.0	0	0.0
18	0	0.0	0	0.0	0	0.0	0	0.0	1	51.5	0	0.0
19	0	0.0	0	0.0	0	0.0	0	0.0	0	0.0	0	0.0

Supplementary Table 2 | Details of strand-coordinate mutagenesis of mutation signatures. For each mutation signature (columns) and processive group length (rows) the table reports (i) the number of groups (ii) the median of the mutation density per Mb across the groups. The mutation density for each group was computed as the number of mutations contained in the group, that is, the processive group length, divided by the size of the group in Mb.

Processive Group Length	Signature 2			Signature 13		
	Lagging	Leading	log10(ratio)	Lagging	Leading	log10(ratio)
1	147,346	115,683	0.105	181,853	140,591	0.112
2	50,935	32,053	0.201	63,734	39,180	0.211
3	19,259	9,919	0.288	25,980	12,564	0.315
4	7,310	3,450	0.326	11,385	4,899	0.366
5	2,856	1,254	0.357	5,083	1,957	0.414
6	1,107	531	0.319	2,566	878	0.465
7	476	210	0.354	1,313	535	0.389
8	244	140	0.240	754	158	0.677
9	134	64	0.317	460	125	0.563
10	39	41	-0.021	223	87	0.406
11	22	0	1.362	205	59	0.536
12	28	8	0.508	87	9	0.944
13	13	0	1.146	55	10	0.707
14	0	0	0.000	50	6	0.862
15	0	0	0.000	47	13	0.535
16	16	0	1.230	60	20	0.463
17	0	0	0.000	16	1	0.929
>3	12,245	5,698	0.332	22,304	8,757	0.406
>4	4,935	2,248	0.341	10,919	3,858	0.452
>5	2,079	994	0.320	5,836	1,901	0.487
>6	972	463	0.322	3,270	1,023	0.505
>7	496	253	0.292	1,957	488	0.603

Processive Group Length	Signature 2			Signature 13		
	Transcribed	Non-Transcribed	log10(ratio)	Lagging	Leading	log10(ratio)
1	66,455	70,795	-0.027	84,148	85,904	-0.009
2	19,654	21,090	-0.031	26,432	26,850	-0.007
3	6,191	6,760	-0.038	8,826	9,366	-0.026
4	2,431	2,517	-0.015	3,834	3,810	0.003
5	803	757	0.026	1,533	1,642	-0.030
6	421	401	0.021	733	869	-0.074
7	119	154	-0.111	345	383	-0.045
8	135	97	0.142	144	184	-0.106
9	44	19	0.352	91	134	-0.167
10	19	31	-0.204	41	59	-0.155
11	0	0	0.000	65	45	0.157
12	30	18	0.213	12	36	-0.454
13	0	0	0.000	4	9	-0.301
14	0	0	0.000	0	0	0.000
15	0	0	0.000	7	8	-0.051
16	0	0	0.000	0	0	0.000
17	0	0	0.000	0	0	0.000
>3	4,002	3,994	0.001	6,809	7,179	-0.023
>4	1,571	1,477	0.027	2,975	3,369	-0.054
>5	768	720	0.028	1,442	1,727	-0.078
>6	347	319	0.037	709	858	-0.083
>7	228	165	0.140	364	475	-0.116

Supplementary Table 3 | Replication and transcriptional strand bias associated with APOBEC-related signatures processivity. The tables report the replication strand (**A**) and the transcription strand (**B**) counts and ratios for the different length of processive groups associated with the APOBEC-related signature 2 and 13. The last five rows report the aggregated counts and ratios (e.g. >3 reports the number of mutations and the respective ratios across all groups composed of more than 3 mutations). From the table, it is clear that the replication strand bias of Signature 13 increases with the length of the processive group. Unlike replication strand, the transcriptional strand bias observed for these signatures does not seem related to the length of the processive group.

Signature	MCF7	BG02ES	BJ	GM06990	GM12801	GM12812	GM12813	GM12878	HeLa-S3	HepG2	HUVEC	IMR90	K562	SK-N-SH
1	0.0145	0.0116	0.0101	0.0104	0.0077	0.0101	0.0122	0.0099	0.0099	0.0119	0.0101	0.0112	0.0077	0.0085
5	0.0137	0.0092	0.0109	0.0120	0.0099	0.0109	0.0125	0.0113	0.0108	0.0127	0.0109	0.0102	0.0104	0.0136
2	0.1473	0.0851	0.0994	0.1147	0.1113	0.1076	0.1125	0.1109	0.1134	0.1284	0.1022	0.1036	0.1006	0.0997
13	0.1634	0.0978	0.1181	0.1301	0.1261	0.1223	0.1286	0.1254	0.1295	0.1474	0.1222	0.1206	0.1162	0.1144
6	-0.0332	-0.0244	-0.0301	-0.0270	-0.0253	-0.0253	-0.0247	-0.0218	-0.0270	-0.0307	-0.0307	-0.0292	-0.0265	-0.0213
20	0.0206	0.0029	0.0124	0.0230	0.0259	0.0223	0.0307	0.0258	0.0265	0.0117	0.0239	0.0192	0.0237	0.0106
26	0.1129	0.0796	0.0876	0.1010	0.0991	0.0987	0.1032	0.0994	0.0980	0.1129	0.0934	0.0894	0.0946	0.0938
3	0.0284	0.0192	0.0257	0.0250	0.0237	0.0235	0.0245	0.0238	0.0261	0.0295	0.0263	0.0270	0.0236	0.0230
8	0.0011	-0.0010	0.0031	-0.0013	-0.0004	0.0012	0.0020	-0.0009	-0.0008	0.0034	0.0004	0.0020	-0.0023	0.0016
18	0.0202	0.0071	0.0106	0.0118	0.0155	0.0141	0.0173	0.0176	0.0165	0.0148	0.0133	0.0170	0.0144	0.0167
17	0.0527	0.0339	0.0439	0.0467	0.0481	0.0484	0.0491	0.0448	0.0475	0.0563	0.0454	0.0489	0.0490	0.0389
30	0.0121	-0.0007	-0.0070	0.0225	-0.0059	0.0036	-0.0031	0.0092	0.0141	0.0038	0.0145	-0.0092	0.0043	0.0057

Supplementary Table 4 | Results of replication strand bias analysis across 14 ENCODE cell lines Replication strand ratio (log10) computed for each signature (row) for 14 different cell lines (column). Negative and positive values are associated with an asymmetry towards the p-to-q leading and p-to-q lagging strand respectively. Results show that the replication strand biases demonstrated by specific signatures are consistent across all the cell lines. Note that MCF-7 exhibits the strongest asymmetry for APOBEC-related signatures 2 (ratio = 0.1473) and 13 (ratio = 0.1634).
